# Venous Thromboembolism in Total Hip and Total Knee Arthroplasty

**DOI:** 10.1001/jamanetworkopen.2023.45883

**Published:** 2023-12-01

**Authors:** Samantha J. Simon, Rushad Patell, Jeffrey I. Zwicker, Dhruv S. Kazi, Brian L. Hollenbeck

**Affiliations:** 1 Research Department, New England Baptist Hospital, Boston, Massachusetts; 2 Division of Hematology and Hematologic Malignancies, Beth Israel Deaconess Medical Center, Boston, Massachusetts; 3 Richard A. and Susan F. Smith Center for Outcomes Research in Cardiology, Beth Israel Deaconess Medical Center, Boston, Massachusetts; 4 Hematology Service, Memorial Sloan Kettering Cancer Center, New York, New York; 5 Harvard Medical School, Boston, Massachusetts

## Abstract

**Importance:**

The optimal pharmacologic thromboprophylaxis agent after total hip and total knee arthroplasty is uncertain and consensus is lacking. Quantifying the risk of postoperative venous thromboembolism (VTE) and bleeding and evaluating comparative effectiveness and safety of the thromboprophylaxis strategies can inform care.

**Objective:**

To quantify risk factors for postoperative VTE and bleeding and compare patient outcomes among pharmacological thromboprophylaxis agents used after total hip and knee arthroplasty.

**Design, Setting, and Participants:**

This retrospective cohort study used data from a large health care claims database. Participants included patients in the United States with hip or knee arthroplasty and continuous insurance enrollment 3 months prior to and following their surgical procedure. Patients were excluded if they received anticoagulation before surgery, received no postsurgical pharmacological thromboprophylaxis, or had multiple postsurgery thromboprophylactic agents. In a propensity-matched analysis, patients receiving a direct oral anticoagulant (DOAC) were matched with those receiving aspirin.

**Exposures:**

Aspirin, apixaban, rivaroxaban, enoxaparin, or warfarin.

**Main Outcomes and Measures:**

The primary outcome was 30-day cumulative incidence of postdischarge VTE. Other outcomes included postdischarge bleeding.

**Results:**

Among 29 264 patients included in the final cohort, 17 040 (58.2%) were female, 27 897 (95.2%) had inpatient admissions with median (IQR) length of stay of 2 (1-2) days, 10 948 (37.4%) underwent total hip arthroplasty, 18 316 (62.6%) underwent total knee arthroplasty; and median (IQR) age was 59 (55-63) years. At 30 days, cumulative incidence of VTE was 1.19% (95% CI, 1.06%-1.32%) and cumulative incidence of bleeding was 3.43% (95% CI, 3.22%-3.64%). In the multivariate analysis, leading risk factors associated with increased VTE risk included prior VTE history (odds ratio [OR], 5.94 [95% CI, 4.29-8.24]), a hereditary hypercoagulable state (OR, 2.64 [95% CI, 1.32-5.28]), knee arthroplasty (OR, 1.65 [95% CI, 1.29-2.10]), and male sex (OR, 1.34 [95% CI, 1.08-1.67]). In a propensity-matched cohort of 7844 DOAC-aspirin pairs, there was no significant difference in the risk of VTE in the first 30 days after the surgical procedure (OR, 1.14 [95% CI, 0.82-1.59]), but postoperative bleeding was more frequent in patients receiving DOACs (OR, 1.36 [95% CI, 1.13-1.62]).

**Conclusions and Relevance:**

In this cohort study of patients who underwent total hip or total knee arthroplasty, underlying patient risk factors, but not choice of aspirin or DOAC, were associated with postsurgical VTE. Postoperative bleeding rates were lower in patients prescribed aspirin. These results suggest that thromboprophylaxis strategies should be patient-centric and tailored to individual risk of thrombosis and bleeding.

## Key Points

**Question:**

Are thromboprophylactic agents and patient risk factors associated with venous thromboembolism (VTE) and bleeding rates after lower extremity arthroplasty?

**Findings:**

In this cohort study of 29 264 patients who underwent total hip arthroplasty or total knee arthroplasty, VTE occurred in 1.19% and bleeding in 3.43% at 30 days. A history of VTE or hereditary hypercoagulable state was associated with a higher rate of VTE.

**Meaning:**

VTE risk following arthroplasty was primarily associated with underlying patient risk factors; these results suggest that thromboprophylaxis strategies should be patient-centric and tailored to individual risk of thrombosis and bleeding.

## Teaser

This cohort study assesses risk factors for postoperative venous thromboembolism and bleeding among patients receiving thromboprophylactic agents after total hip and knee arthroplasty.

## Introduction

An estimated 1.5 million lower extremity arthroplasty procedures are performed in the United States annually.^1^ Deep vein thrombosis (DVT) and pulmonary embolism (PE) occur in 0.6% to 3.0% of total hip arthroplasty (THA) and total knee arthroplasty (TKA) cases^2,3^ and contribute to postoperative mortality, morbidity, and health care spending.^4,5,6,7,8,9,10^ Venous thromboembolism (VTE) risk is determined by patient demographics, comorbidities, and thromboprophylaxis strategy.^11,12,13,14^ Although pharmacologic thromboprophylaxis strategies in orthopedic patients is often procedure specific, underlying risk factors that mediate thrombotic and bleeding risk can make decision-making more nuanced.^15^

Different thromboprophylaxis strategies to reduce postoperative VTE after THA or TKA have been proposed, with variable levels of supporting literature.^2,16,17,18,19^ All thromboprophylaxis medications have potential for postoperative bleeding, especially in patients with additional risk factors for hemorrhage.^20,21,22^ The optimal choice and selection of thromboprophylaxis in patients after THA or TKA remains unclear and consensus among clinical societies is lacking.^23,24,25,26,27^ In particular, how aspirin compares with more contemporary prophylactic anticoagulants is not well established. Regardless, the use of aspirin after THA or TKA in the Unites States is frequent, although the lack of comparative data are acknowledged.^28,29,30^

We analyzed a national health care claims–based database to compare the incidence of VTE and bleeding risk after THA or TKA by underlying risk factors. In a propensity-matched approach we further compared the risk of postoperative VTE and bleeding with the use of direct oral anticoagulants (DOACs) and aspirin.

## Methods

### MarketScan Database

This cohort study used data from the MarketScan Commercial Claims and Encounters databases and MarketScan Medicare Supplemental and Coordination of Benefits databases from January 1, 2017, to December 31, 2019. It includes coverage of the United States and uses private health insurance claims and billing codes to report data on more than 245 million patients. The Medicare supplemental database was used to include those with health care that is employer-paid Medicare.

### Study Population

The study was reviewed and deemed exempt by the New England Baptist Hospital institutional review board, thus informed consent was not needed in accordance with 45 CFR §46. We identified patients undergoing primary THA or TKA between March 1, 2017, and September 30, 2019, using *Current Procedural Terminology* codes (THA: 27130; TKA: 27447). This study followed the Strengthening the Reporting of Observational Studies in Epidemiology (STROBE) reporting guideline for cohort studies. The study cohort included patients with continuous insurance enrollment 3 months prior to and following arthroplasty. Among patients who had contralateral surgical procedures during the study period, only the first procedure was included. To ensure we only included patients receiving anticoagulants or antiplatelets for thromboprophylaxis, we excluded patients prescribed an anticoagulant medication 14 to 90 days prior to the surgical procedure. Patients prescribed greater than 1 or no postoperative thromboprophylactic medication were also excluded.

### Comorbidities

We identified the following comorbidities (coded up to 36 months prior to the index surgical procedure) based on likelihood to influence risk of postsurgical thrombotic and bleeding risk using appropriate *International Statistical Classification of Diseases and Related Health Problems, Tenth Revision (ICD-10)* codes: obesity, chronic kidney disease (CKD), antiphospholipid antibody, lupus anticoagulant, history of VTE, factor V Leiden, antithrombin deficiency, prothrombin gene mutation, protein C or S deficiency, and cancer (eTable 1 in Supplement 1).^5^

### Pharmacological Thromboprophylaxis

We collected data on thromboprophylactic medication using national drug codes for prescription aspirin, rivaroxaban, apixaban, warfarin, and enoxaparin (eTable 2 in Supplement 1). We used the first prescription for a thromboprophylactic medication between 10 days prior to the index surgical procedure, up to 14 days following the procedure. Aspirin prescriptions included 81 mg and 325 mg both once and twice a day.

### Outcomes

The primary outcome was cumulative incidence of VTE (including postdischarge DVT and PE) at 30 days.^8,16^ Secondary outcomes were 90-day cumulative incidence of VTE and 30-day and 90-day postdischarge bleeding events. The *ICD-10* codes used to identify these VTE (including lower extremity DVT and PEs) and bleeding events (including intracranial, intraocular, gastrointestinal, genitourinary, and intraarticular bleeding) in the claims data were modified from previously validated studies (eTable 3 and eTable 4 in Supplement 1).^19,31,32,33^

### Statistical Analysis

#### Main Analysis

Cumulative incidences of VTE and bleeding were calculated individually without censoring for other outcomes. We performed univariate χ^2^ and regression analysis to identify risk factors and compare thromboprophylactic agents for 30-day VTE events. Statistical significance was set at 2-sided *P* < .05. Multivariable logistic regression was performed to identify independent risk factors for the 30-day VTE or bleeding events, adjusting for sex, age, THA or TKA, length of stay (LOS), obesity, cancer, CKD, antiphospholipid antibody or lupus anticoagulant, history of VTE, and a hereditary hypercoagulable state (factor V Leiden, antithrombin deficiency, prothrombin gene mutation, protein C deficiency, or protein S deficiency). We included LOS as a surrogate for uncontrolled comorbidities, complexity of surgical procedure, or immediate postoperative complications. Data are presented as odds ratios (OR) with 95% CI. Statistical analysis was performed using SAS software version 9.4 (SAS Institute) from December 7, 2021, to September 23, 2023.

Propensity score matching was done to compare bleeding and thrombotic outcomes in patients prescribed aspirin or DOACs. We included only rivaroxaban and apixaban in the DOAC group as other DOACs were infrequent. Propensity for receiving a DOAC was calculated for each patient based on age, sex, year procedure was performed, inpatient or outpatient arthroplasty, LOS, THA or TKA, hereditary hypercoagulable state, obesity, CKD, cancer, antiphospholipid antibody or lupus anticoagulant, and history of VTE. Patients receiving aspirin or DOAC were matched in 1:1 ratio, with the allowable difference in propensity scores set at 0.01% using greedy nearest neighbor matching without replacement. Standardized mean differences (SMDs) were calculated to compare the matched groups. We calculated 30-day and 90-day cumulative incidence of bleeding and VTE for the aspirin and DOAC groups. We also calculated the OR and 95% CI on the matched data for primary and secondary outcomes.

#### Sensitivity Analyses

We performed sensitivity analyses to assess the robustness of our findings in the primary analyses. E-values (defined as the minimum strength of association that an unmeasured confounder would need to have to fully explain away a specific association, conditional on the measured covariates) were calculated for VTE ORs.^34,35,36^ To assess for residual confounding after propensity score matching, we compared bleeding rates 90 days prior to surgical procedure. To detect potential bias, we compared the rates of negative (unrelated) control outcomes, cholecystitis and motor vehicle accidents, between the aspirin and DOAC groups in the matched analysis.

#### Secondary Analyses

To further refine our definition of VTE in our matched cohort, we performed the following analysis: 30-day PE rates, odds of VTE in the 30- to 60-day and 60- to 90-day postoperative windows, VTE rates in THA and TKA separately, and VTE and bleeding cumulative incidences stratified by aspirin doses.

## Results

### Patient Overview

Of the 132 237 patients who underwent THA or TKA between 2017 and 2019 and had continuous insurance enrollment, we excluded 6688 patients with chronic anticoagulation, 774 patients prescribed at least 2 anticoagulants, and 95 511 patients with no medication prescription recorded. Among 29 264 patients with complete data included in the final cohort, 17 040 (58.2%) were female, 27 897 (95.2%) had inpatient admissions with median (IQR) LOS of 2 (1-2) days, 10 948 (37.4%) had THA; and median (IQR) age was 59 (55-63) years (Table). Common comorbidities included 8212 patients with obesity (28.1%), 1142 with CKD (3.9%), and 1328 with cancer (4.5%). Missing data accounted for a small percentage overall. For example, 7 patients (<0.1%) did not have location of surgical procedure coded, and for these patients we assumed that if their LOS was greater than 0 days, then they had inpatient surgical procedures, otherwise they had outpatient surgical procedures.

**Table.** Overview of Patient Characteristics, Surgical Procedure Variables, and Thromboprophylactic Medication

Variables	Patients, No. (%) (N = 29 264)
Age, y	
<65	23 952 (81.9)
≥65	5312 (18.2)
Sex	
Male	12 224 (41.8)
Female	17 040 (58.2)
Comorbidities	
History of venous thromboembolism	731 (2.5)
Obesity	8212 (28.1)
Cancer	1328 (4.5)
Chronic kidney disease	1142 (3.9)
Antiphospholipid antibody or lupus anticoagulant	255 (0.9)
Hereditary hypercoagulable state	150 (0.5)
Surgical procedure	
Total hip arthroplasty	10 948 (37.4)
Total knee arthroplasty	18 316 (62.6)
Location of surgical procedure	
Outpatient (hospital-based)	1247 (4.3)
Inpatient	27 897 (95.3)
Ambulatory surgical center	120 (0.4)
Year of surgical procedure	
2017	11 350 (38.8)
2018	11 481 (39.2)
2019	6433 (22.0)
Length of stay, d	
0	4731 (16.2)
1-2	9318 (31.8)
3-4	14 395 (49.2)
≥5	820 (2.8)
Thromboprophylactic medication	
Aspirin	10 082 (34.5)
Enoxaparin	5764 (19.7)
Rivaroxaban	7068 (24.2)
Warfarin	3097 (10.6)
Apixaban	3253 (11.1)

### Use of Thromboprophylaxis Medications

Aspirin was prescribed most frequently to 10 082 patients (34.5%), followed by 7068 (24.2%) receiving rivaroxaban, 5764 (19.7%) receiving enoxaparin, 3253 (11.1%) receiving apixaban, and 3097 (10.6%) receiving warfarin (Table). The median (IQR) durations of prescriptions were 31 (31-32) days for aspirin, 17 (14-31) days for rivaroxaban, 15 (12-22) days for enoxaparin, 18 (14-31) days for apixaban, and 31 (24-33) days for warfarin.

### VTE and Bleeding Rates

**Figure 1.**
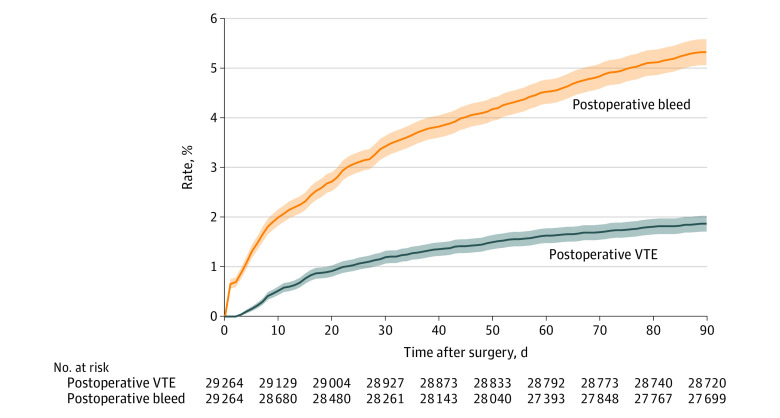
Cumulative Incidence of Venous Thromboembolism (VTE) and Bleeding 0 to 90 Days After Surgical Procedure Figure shows the cumulative incidence of VTE and bleeding 0 to 90 days after total hip arthroplasty and total knee arthroplasty surgical procedures. Shaded areas represent 95% CIs.

The cumulative incidence of VTE was 1.19% (95% CI, 1.06-1.32%) at 30 days and 1.86% (95% CI, 1.70%-2.02%) at 90 days (Figure 1). The cumulative incidence of bleeding was 3.43% (95% CI, 3.22%-3.64%) at 30 days and 5.33% (95% CI, 5.07%-5.59%) at 90 days. When stratified by the presence of a thrombotic risk factor (including obesity, cancer, CKD, history of VTE, antiphospholipid antibody or lupus anticoagulant, and hereditary hypercoagulable state), the cumulative incidence of VTE remained higher through the 90-day postoperative period for those with a prothrombotic risk factor (2.45% [95% CI, 2.15%-2.75%]) than for those without a thrombotic risk factor (1.53% [95% CI, 1.35%-1.71%]).

### VTE Risk Factors

**Figure 2.**
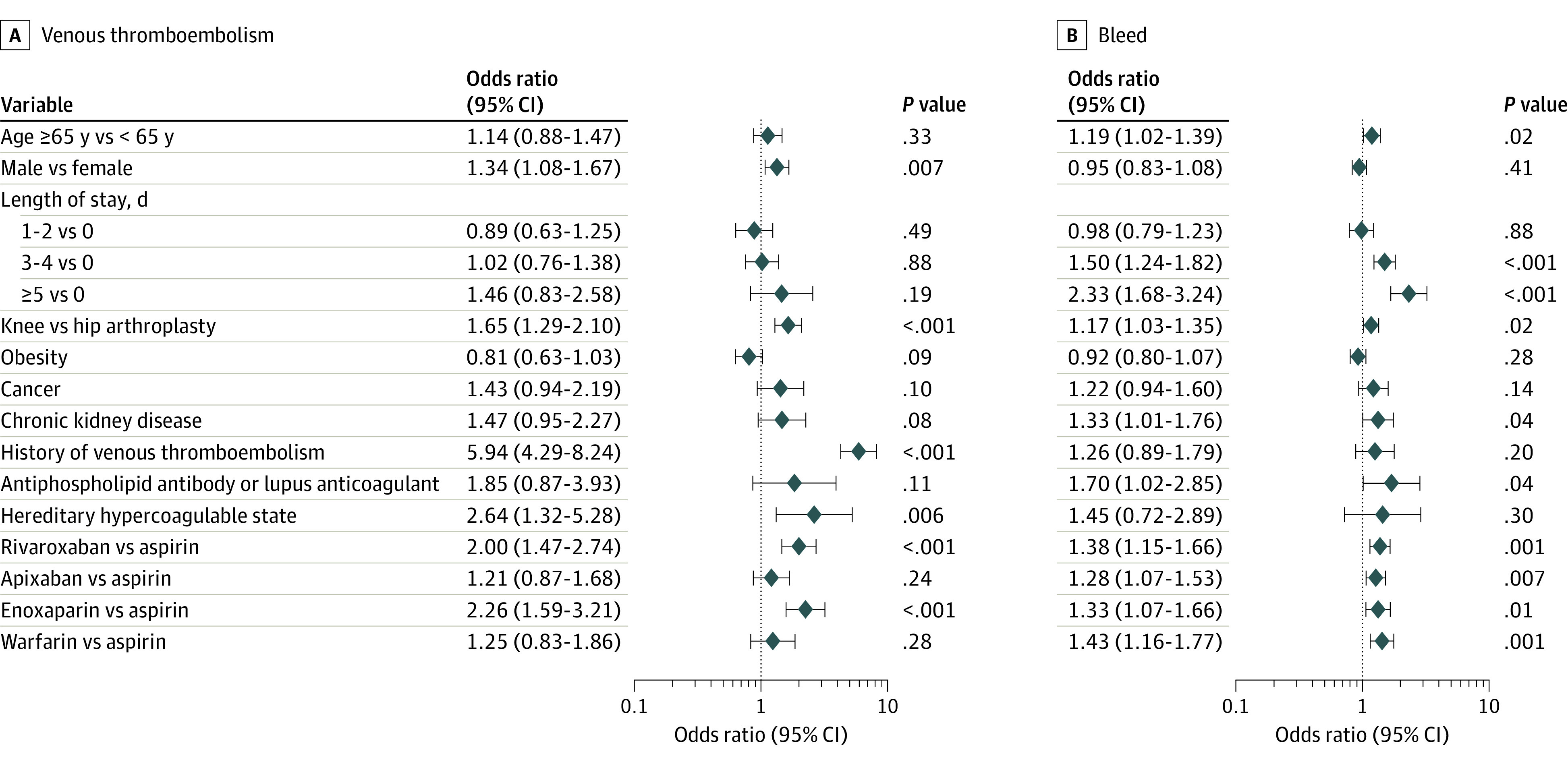
Multivariable Analysis of 30-Day Postoperative Venous Thromboembolism and Bleeding Figure shows odds of developing a venous thromboembolism and odds of developing a bleed.

In the univariate analysis, hereditary hypercoagulable state and history of thrombosis had the highest unadjusted VTE risk (eTable 5 in Supplement 1). On multivariate analysis, factors associated with significant higher odds of 30-day VTE included a history of VTE (OR, 5.94 [95% CI, 4.29-8.24]), hereditary hypercoagulable state (OR, 2.64 [95% CI, 1.32-5.28]), TKA (OR, 1.65 [95% CI, 1.29-2.10]), and male sex (OR, 1.34 [95% CI, 1.08-1.67]) (Figure 2). For 30-day bleeds, an LOS of at least 3 days, antiphospholipid antibody or lupus anticoagulant, CKD, age greater than or equal to 65 years, and TKA were associated with increased odds of a postoperative bleed.

For 30-day VTE, adjusted for patient and surgical risk factors, patients prescribed aspirin had lower VTE rates when compared with those prescribed enoxaparin or rivaroxaban. There was no statistically significant difference in 30-day VTE rates between those prescribed aspirin and those prescribed warfarin or apixaban. Aspirin was associated with less bleeding than other agents. Multivariable analysis for 90-day outcomes also favored aspirin as having lower VTE and bleeding rates (eTable 6 in Supplement 1).

### Propensity-Matched Analysis

In the unmatched cohort, patients prescribed DOACs had higher incidences of VTE risk factors, including history of VTE, CKD, and hereditary hypercoagulable state. This was associated with the higher median (IQR) propensity score in the DOAC group (0.54 [0.42-0.61]) compared with the aspirin group (0.44 [0.40-0.59]) (eFigure 1 in Supplement 1).

The propensity-matched analysis included 15 688 patients (7844 matched pairs; eTable 7 in Supplement 1). Propensity for receiving a DOAC ranged from 32.1% to 94.5% in the matched cohort. Prior to matching, absolute SMDs between the DOAC and aspirin group ranged from less than 0.01 to 0.19. After matching, the absolute SMDs for all variables decreased to 0.00, eliminating detectable differences in risk factors between the groups (eFigure 2 in Supplement 1).

**Figure 3.**
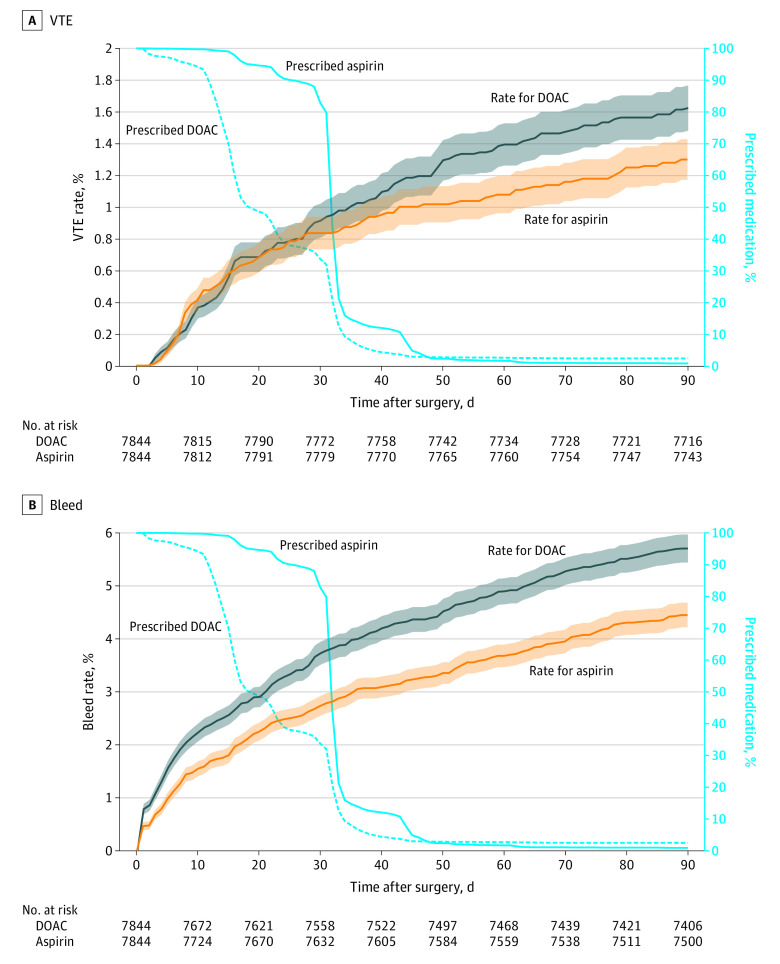
Cumulative Incidence of Venous Thromboembolism (VTE) and Bleeding 0 to 90 Days After Surgical Procedure in a Propensity-Matched Cohort Shaded areas represent 95% CIs. DOAC indicates direct oral anticoagulants.

Thromboprophylaxis prescription duration was assessed in comparison with the occurrence of VTE and bleeding events (Figure 3). In our matched cohort the median (IQR) duration a patient was prescribed was 31 (31-32) days for aspirin and 18 (14-31) days for DOACs; 43.6% (3189 of 7844) of the aspirin group and 20.6% (1616 of 7844) of the DOAC group were prescribed medication for more than 31 days.

In the propensity-matched cohort, 30-day VTE cumulative incidences were 0.92% (95% CI, 0.83%-1.05%) for the DOAC group and 0.83% (95% CI, 0.73%-0.93%) for the aspirin group. At 90 days, the cumulative incidence was 1.63% (95% CI, 1.49%-1.77%) for the DOAC group and 1.29% (95% CI, 1.16%-1.42%) for the aspirin group.

Patients receiving a DOAC had similar odds of VTE at 30 days compared with patients receiving aspirin (OR, 1.14 [95% CI, 0.82-1.59]), but odds of bleeding were higher in the DOAC group (OR, 1.36 [95% CI, 1.13-1.62]). Similarly, at 90 days, the odds of VTE were not statistically different between the DOAC and aspirin groups (OR, 1.28 [95% CI, 0.98-1.66]), but odds of bleeding were higher in patients receiving DOACs (OR, 1.27 [95% CI, 1.10-1.47]). The only difference in odds of VTE was seen between days 30 to 60 where the odds of VTE were higher in the DOAC group compared with the aspirin group (OR, 1.89 [95% CI, 1.09-3.30]).

### Sensitivity Analyses

The E-values calculated for the 30-day VTE OR of the 2 statistically significant prophylactic medications were 3.95 for enoxaparin and 3.41 for rivaroxaban. This was higher than any OR calculated for the known risk factors of 30-day VTE, with the exception of history of VTE.

For the propensity-matched patients, the bleeding rates 90 days prior to surgical procedure were not statistically significant between the aspirin and DOAC groups. Additionally, there was no statistical difference in the incidence of the 2 negative control outcomes, cholecystitis and motor vehicle accidents, between the aspirin and DOAC groups.

### Secondary Analyses

The 30-day cumulative incidence of PE was not statistically significant between the aspirin and DOAC groups; and out of the matched cohort, patients who received THA alone and patients who received TKA alone both showed no difference in 30-day VTE rates. When stratifying by aspirin doses, there was no difference in 30-day VTE or bleeding rates.

## Discussion

In this cohort study, the 30-day cumulative incidence for VTE after THA or TKA was low at 1.19%, with higher rates in patients with thrombotic risk factors. Aspirin was a common VTE thromboprophylaxis choice and was not associated with higher rates of VTE compared with other anticoagulants, however, rates of bleeding were significantly lower with aspirin. Furthermore, in the propensity-matched analysis, aspirin was shown to have a potentially longer lasting effect as odds of VTE were lower in the 30- to 60-day postoperative window with lower overall VTE rates at 90 days compared with patients receiving DOAC.

A similar study that used the same database from an older timeframe (2004-2013) demonstrated 6-month DVT rates ranging from 3.12% to 3.42% in a comparable population.^3^ This may reflect lower incidence of VTE after orthopedic surgical procedures at a population level over the last decade due to secular trends in management practices (eg, shorter LOS, outpatient arthroplasty, and earlier mobilization) or increasing use of DOACs. A recent report that included 363 530 patients who underwent THA or TKA from 2008 to 2016 in a national surgical quality database demonstrated an overall VTE rate of 0.6% to 1.4%, similar to rates observed in the current study.^37^ Acquired and hereditary conditions can increase thrombotic risk in patients through different mechanisms, and may be additive.^38,39^ In this large claims-based study, the risk remained statistically significant for male vs female, TKA vs THA, and known thrombotic risk factors.^11,12,13^

Over the last decade, aspirin has become common for thromboprophylaxis after THA or TKA.^16,17,18,19,40^ In contemporary trials, aspirin showed noninferiority compared with low-molecular-weight heparin or rivaroxaban in reducing VTE in THA and TKA.^17,41^ However, a separate randomized clinical trial (RCT) that compared aspirin with enoxaparin in reducing symptomatic VTE after THA or TKA failed to meet noninferiority criteria for aspirin.^42^ A comparative clinical trial with aspirin and DOACs is ongoing.^43^ In this study based on clinical data, patients prescribed aspirin appear to have similar postoperative VTE rates and significantly less bleeding compared with patients prescribed DOACs, consistent with recent studies.^8,17,18,19^ While the effectiveness of aspirin when used as sole prophylaxis compared with DOACs has not been fully resolved, the existing evidence, convenience, and perceived lower bleeding risk have made aspirin VTE prophylaxis widespread in recent years.^29^

Thromboprophylaxis selection involves balancing thrombotic risk with bleeding risk. We found aspirin was associated with significantly lower bleeding risk than other anticoagulants prescribed after THA and TKA. These results are supported by data from RCTs that have compared aspirin with other anticoagulants. A systematic review found a lower rate of bleeding with aspirin compared with anticoagulants after hip fracture repair and a trend toward lower rates of bleeding after lower extremity arthroplasty.^44^ In contrast, a recent meta-analysis that included 13 RCTs showed no difference in rates of bleeding complications.^18^ In our study, the favorable bleeding profile for aspirin may be drawn from a large population of patients who could be at higher risk of bleeding than the carefully selected participants of RCTs. Clinical data can support relative effectiveness and safety, which supplements efficacy data from well-designed randomized studies.^45,46^

In addition to lower bleeding risk, our study found that the aspirin group had lower odds of VTE during days 30 to 60, which is the period when most patients discontinue prophylaxis. This illustrates how aspirin may continue to reduce VTE risk after the patient is no longer taking the medication. This could be by way of the inhibition of cyclooxygenase isoenzymes by aspirin,^47^ which irreversibly inhibits the action of platelets, thus extending the effect equal to the life of the platelet.^48^

### Limitations

This study had limitations. Although the use of an insurance database allowed us to capture a large sample that is reflective of patient populations and circumstances, it is inherently limited by the quality of billing information. We used previously validated algorithms for the outcome measurements to minimize biases introduced through inaccurate coding,^19,31,32,33^ however, there may be differences in strategies for diagnosing thrombotic events that could be influenced by the pharmacologic thromboprophylaxis which could influence the results. Additionally, national drug codes are unable to capture information about over-the-counter medications and reflect only what a patient was prescribed, not necessarily what was taken. Therefore, we could not verify that patients without a prescription did not actually receive over-the-counter aspirin for prophylaxis or that patients prescribed DOACs were also taking aspirin. We applied exclusion criteria to ensure we analyzed patients with sufficient data (including the absence of a script for thromboprophylaxis); however, it is notable that ultimately approximately 25% of the originally identified population were included. The MarketScan database includes only privately insured patients, thus the population in this study is younger than those enrolled in clinical trials.^18,49^ Therefore, data presented in this study may not be as generalizable to an older patient population. Retrospective studies are susceptible to residual confounding. For example, although we included several known acquired and hereditary risk factors for thrombosis and bleeding in the models, comorbidities such as CKD can influence the choice of pharmacologic thoromboprophylaxis as well as be associated with increased risk of thrombosis and bleeding. Similar rates of bleeding in the 90 days prior and similar rates of falsification end points (cholecystitis and motor vehicle accidents) in the 90 days after index surgical procedure suggest that the groups were adequately matched. We also calculated E-values to assess the presence of potential but unknown confounders. The ORs for such unknown confounders were shown to be greater than 3.0 (higher than most of the known risk factors included), which makes it unlikely that such unknown confounders exist. We note that the variability in the median prescription length of aspirin compared with DOACs could be an important contributing factor to the findings, however, we feel these data reflect clinical practice patterns and are thus important to elucidate.

## Conclusions

In this cohort study of patients who received THA or TKA, we found that the risk of VTE was associated with underlying risk factors and preexisting diagnoses, rather than choice of thromboprophylactic medication. A propensity-matched subset of patients that compared aspirin with DOACs had equivalent rates of VTE at 30 days, but lower VTE rates at 90 days and lower bleeding rates. These results suggest a need for patient-centric thromboprophylaxis strategies tailored to individual risk of thrombosis and bleeding.

## Notes

Supplement 1. **eTable 1.** International Classification of Diseases-10th Revision (ICD-10) Codes for Comorbidities **eTable 2.** National Drug Codes **eTable 3.** International Classification of Diseases-10th Revision (ICD-10) Codes for Venous Thromboembolism **eTable 4.** International Classification of Diseases-10th Revision (ICD-10) Codes for Bleed **eTable 5.** Chi-Squared Univariate Analysis of 30-Day Post-Operative Venous Thromboembolism (VTE) **eTable 6.** Multivariable Analysis of 90-Day Post-Operative Venous Thromboembolism and Bleeding **eFigure 1.** Box Plot of Propensity Scores for Receiving a Direct Oral Anticoagulant (DOAC) by DOAC and Aspirin Prior to Matching **eTable 7.** Overview of Patient and Surgical Variables of the Propensity Matched Cohort **eFigure 2.** Absolute Standardized Mean Differences Between Direct Oral Anticoagulant and Aspirin Patients

Supplement 2.
Data Sharing Statement

## References

[1] Singh JA, Yu S, Chen L, Cleveland JD. Rates of total joint replacement in the United States: future projections to 2020-2040 using the national inpatient sample. J Rheumatol. 2019;46(9):1134-1140. doi:10.3899/jrheum.170990 30988126

[2] Oberweis BS, Cuff G, Rosenberg A, . Platelet aggregation and coagulation factors in orthopedic surgery. J Thromb Thrombolysis. 2014;38(4):430-438. doi:10.1007/s11239-014-1078-1 24874897

[3] Bawa H, Weick JW, Dirschl DR, Luu HH. Trends in deep vein thrombosis prophylaxis and deep vein thrombosis rates after total hip and knee arthroplasty. J Am Acad Orthop Surg. 2018;26(19):698-705. doi:10.5435/JAAOS-D-17-00235 30153117

[4] Coventry MB, Nolan DR, Beckenbaugh RD. “Delayed” prophylactic anticoagulation: a study of results and complications in 2,012 total hip arthroplasties. J Bone Joint Surg Am. 1973;55(7):1487-1492. doi:10.2106/00004623-197355070-00016 4758718

[5] Friedman RJ, Sengupta N, Lees M. Economic impact of venous thromboembolism after hip and knee arthroplasty: potential impact of rivaroxaban. Expert Rev Pharmacoecon Outcomes Res. 2011;11(3):299-306. doi:10.1586/erp.11.15 21671699

[6] Fisher WD. Impact of venous thromboembolism on clinical management and therapy after hip and knee arthroplasty. Can J Surg. 2011;54(5):344-351. doi:10.1503/cjs.007310 21774881 PMC3195664

[7] Xu K, Chan NC, Ibrahim Q, . Reduction in mortality following elective major hip and knee surgery: a systematic review and meta-analysis. Thromb Haemost. 2019;119(4):668-674. doi:10.1055/s-0039-1677732 30699447

[8] Lanes S, Fraeman K, Meyers A, Wood Ives J, Huang HY. Incidence rates for thromboembolic, bleeding and hepatic outcomes in patients undergoing hip or knee replacement surgery. J Thromb Haemost. 2011;9(2):325-332. doi:10.1111/j.1538-7836.2010.04155.x 21129148

[9] Khorana AA, Kuderer NM, McCrae K, . Cancer associated thrombosis and mortality in patients with cancer stratified by khorana score risk levels. Cancer Med. 2020;9(21):8062-8073. doi:10.1002/cam4.3437 32954653 PMC7643641

[10] Santana DC, Emara AK, Orr MN, . An update on venous thromboembolism rates and prophylaxis in hip and knee arthroplasty in 2020. Medicina (Kaunas). 2020;56(9):E416. doi:10.3390/medicina56090416 32824931 PMC7558636

[11] Zhang ZH, Shen B, Yang J, Zhou ZK, Kang PD, Pei FX. Risk factors for venous thromboembolism of total hip arthroplasty and total knee arthroplasty: a systematic review of evidences in ten years. BMC Musculoskelet Disord. 2015;16(1):24. doi:10.1186/s12891-015-0470-0 25887100 PMC4328702

[12] Zhang J, Chen Z, Zheng J, Breusch SJ, Tian J. Risk factors for venous thromboembolism after total hip and total knee arthroplasty: a meta-analysis. Arch Orthop Trauma Surg. 2015;135(6):759-772. doi:10.1007/s00402-015-2208-8 25854654

[13] White RH, Henderson MC. Risk factors for venous thromboembolism after total hip and knee replacement surgery. Curr Opin Pulm Med. 2002;8(5):365-371. doi:10.1097/00063198-200209000-00004 12172437

[14] Falck-Ytter Y, Francis CW, Johanson NA, . Prevention of VTE in orthopedic surgery patients: antithrombotic therapy and prevention of thrombosis, 9th ed: American College of Chest Physicians evidence-based clinical practice guidelines. Chest. 2012;141(2 Suppl):e278S-e325S. doi:10.1378/chest.11-2404 22315265 PMC3278063

[15] Kahn SR, Shivakumar S. What’s new in VTE risk and prevention in orthopedic surgery. Res Pract Thromb Haemost. 2020;4(3):366-376. doi:10.1002/rth2.12323 32211571 PMC7086463

[16] Agaba P, Kildow BJ, Dhotar H, Seyler TM, Bolognesi M. Comparison of postoperative complications after total hip arthroplasty among patients receiving aspirin, enoxaparin, warfarin, and factor Xa inhibitors. J Orthop. 2017;14(4):537-543. doi:10.1016/j.jor.2017.08.002 28878512 PMC5574820

[17] Anderson DR, Dunbar M, Murnaghan J, . Aspirin or rivaroxaban for VTE prophylaxis after hip or knee arthroplasty. N Engl J Med. 2018;378(8):699-707. doi:10.1056/NEJMoa1712746 29466159

[18] Matharu GS, Kunutsor SK, Judge A, Blom AW, Whitehouse MR. Clinical effectiveness and safety of aspirin for venous thromboembolism prophylaxis after total hip and knee replacement: a systematic review and meta-analysis of randomized clinical trials. JAMA Intern Med. 2020;180(3):376-384. doi:10.1001/jamainternmed.2019.6108 32011647 PMC7042877

[19] Shah S, Norby FL, Datta YH, . Comparative effectiveness of direct oral anticoagulants and warfarin in patients with cancer and atrial fibrillation. Blood Adv. 2018;2(3):200-209. doi:10.1182/bloodadvances.2017010694 29378726 PMC5812321

[20] Kleiboer B, Layer MA, Cafuir LA, . Postoperative bleeding complications in patients with hemophilia undergoing major orthopedic surgery: a prospective multicenter observational study. J Thromb Haemost. 2022;20(4):857-865. doi:10.1111/jth.15654 35080347 PMC8940712

[21] Carling MS, Jeppsson A, Eriksson BI, Brisby H. Transfusions and blood loss in total hip and knee arthroplasty: a prospective observational study. J Orthop Surg Res. 2015;10(1):48. doi:10.1186/s13018-015-0188-6 25889413 PMC4383080

[22] Spiezia L, Vasques F, Behr A, . Perioperative coagulation assessment of patients undergoing major elective orthopedic surgery. Intern Emerg Med. 2016;11(6):793-801. doi:10.1007/s11739-016-1414-x 26951189

[23] Anderson DR, Morgano GP, Bennett C, . American Society of Hematology 2019 guidelines for management of venous thromboembolism: prevention of venous thromboembolism in surgical hospitalized patients. Blood Adv. 2019;3(23):3898-3944. doi:10.1182/bloodadvances.2019000975 31794602 PMC6963238

[24] Mont MA, Jacobs JJ, Boggio LN, ; AAOS. Preventing venous thromboembolic disease in patients undergoing elective hip and knee arthroplasty. J Am Acad Orthop Surg. 2011;19(12):768-776. doi:10.5435/00124635-201112000-00007 22134209

[25] Gee E. The National VTE Exemplar Centres Network response to implementation of updated NICE guidance: venous thromboembolism in over 16s: reducing the risk of hospital-acquired deep vein thrombosis or pulmonary embolism (NG89). Br J Haematol. 2019;186(5):792-793. doi:10.1111/bjh.16010 31168834

[26] The ICM-VTE Hip & Knee Delegates. Recommendations from the ICM-VTE: hip & knee. J Bone Joint Surg Am. 2022;104(suppl 1):180-231. doi:10.2106/JBJS.21.01529 35315610

[27] Muscatelli SR, Charters MA, Hallstrom BR. Time for an update? a look at current guidelines for venous thromboembolism prophylaxis after hip and knee arthroplasty and hip fracture. Arthroplast Today. 2021;10:105-107. doi:10.1016/j.artd.2021.06.015 34337116 PMC8318891

[28] Azboy I, Barrack R, Thomas AM, Haddad FS, Parvizi J. Aspirin and the prevention of venous thromboembolism following total joint arthroplasty: commonly asked questions. Bone Joint J. 2017;99-B(11):1420-1430. doi:10.1302/0301-620X.99B11.BJJ-2017-0337.R2 29092979 PMC5742873

[29] Baumgartner C, Maselli J, Auerbach AD, Fang MC. Aspirin compared with anticoagulation to prevent venous thromboembolism after knee or hip arthroplasty: a large retrospective cohort study. J Gen Intern Med. 2019;34(10):2038-2046. doi:10.1007/s11606-019-05122-3 31236894 PMC6816584

[30] Prevention of pulmonary embolism and deep vein thrombosis with low dose aspirin: pulmonary embolism prevention (PEP) trial. Lancet. 2000;355(9212):1295-1302. doi:10.1016/S0140-6736(00)02110-3 10776741

[31] Lawrence K, Joos C, Jones AE, Johnson SA, Witt DM. Assessing the accuracy of ICD-10 codes for identifying acute thromboembolic events among patients receiving anticoagulation therapy. J Thromb Thrombolysis. 2019;48(2):181-186. doi:10.1007/s11239-019-01885-y 31124033

[32] Lutsey PL, Zakai NA, MacLehose RF, . Risk of hospitalised bleeding in comparisons of oral anticoagulant options for the primary treatment of venous thromboembolism. Br J Haematol. 2019;185(5):903-911. doi:10.1111/bjh.15857 30919942 PMC6536346

[33] Molander V, Bower H, Askling J. Validation and characterization of venous thromboembolism diagnoses in the Swedish National Patient Register among patients with rheumatoid arthritis. Scandinavian Journal of Rheumatology. Published online January 13, 2022. doi:10.1080/03009742.2021.2001907 35023437

[34] VanderWeele TJ, Ding P. Sensitivity analysis in observational research: introducing the E-Value. Ann Intern Med. 2017;167(4):268-274. doi:10.7326/M16-2607 28693043

[35] Mathur MB, Ding P, Riddell CA, VanderWeele TJ. Website and R package for computing E-values. Epidemiology. 2018;29(5):e45-e47. doi:10.1097/EDE.0000000000000864 29912013 PMC6066405

[36] Blum MR, Tan YJ, Ioannidis JPA. Use of e-values for addressing confounding in observational studies-an empirical assessment of the literature. Int J Epidemiol. 2020;49(5):1482-1494. doi:10.1093/ije/dyz261 31930286

[37] Warren JA, Sundaram K, Anis HK, Kamath AF, Higuera CA, Piuzzi NS. Have venous thromboembolism rates decreased in total hip and knee arthroplasty? J Arthroplasty. 2020;35(1):259-264. doi:10.1016/j.arth.2019.08.049 31530463

[38] Centers for Disease Control and Prevention. Data and statistics on venous thromboembolism. Published June 9, 2022. https://www.cdc.gov/ncbddd/dvt/data.html

[39] Senst B, Tadi P, Basit H, Jan A. Hypercoagulability. In: StatPearls. StatPearls Publishing; 2022. Accessed July 28, 2022. https://www.ncbi.nlm.nih.gov/books/NBK538251/30855839

[40] Abdel MP, Meneghini RM, Berry DJ. Current practice trends in primary hip and knee arthroplasties among members of the American Association of Hip and Knee Surgeons: an update during the COVID-19 pandemic. J Arthroplasty. 2021;36(7S):S40-S44.e3. doi:10.1016/j.arth.2021.01.080 33640185

[41] Anderson DR, Dunbar MJ, Bohm ER, . Aspirin versus low-molecular-weight heparin for extended venous thromboembolism prophylaxis after total hip arthroplasty: a randomized trial. Ann Intern Med. 2013;158(11):800-806. doi:10.7326/0003-4819-158-11-201306040-00004 23732713

[42] Sidhu VS, Kelly TL, Pratt N, ; CRISTAL Study Group. Effect of aspirin vs enoxaparin on symptomatic venous thromboembolism in patients undergoing hip or knee arthroplasty: the CRISTAL randomized trial. JAMA. 2022;328(8):719-727. doi:10.1001/jama.2022.13416 35997730 PMC9399863

[43] Pellegrini V, Lambourne C. Comparative Effectiveness of Pulmonary Embolism Prevention After Hip and Knee Replacement (PEPPER). ClinicalTrials.gov identifier: NCT02810704. Updated November 14, 2022. Accessed January 4, 2023. https://clinicaltrials.gov/ct2/show/study/NCT02810704

[44] Drescher FS, Sirovich BE, Lee A, Morrison DH, Chiang WH, Larson RJ. Aspirin versus anticoagulation for prevention of venous thromboembolism major lower extremity orthopedic surgery: a systematic review and meta-analysis. J Hosp Med. 2014;9(9):579-585. doi:10.1002/jhm.2224 25045166

[45] Eichler HG, Pignatti F, Schwarzer-Daum B, . Randomized controlled trials versus real world evidence: neither magic nor myth. Clin Pharmacol Ther. 2021;109(5):1212-1218. doi:10.1002/cpt.2083 33063841 PMC8246742

[46] Bala A, Huddleston JI III, Goodman SB, Maloney WJ, Amanatullah DF. Venous thromboembolism prophylaxis after TKA: aspirin, warfarin, enoxaparin, or factor Xa inhibitors? Clin Orthop Relat Res. 2017;475(9):2205-2213. doi:10.1007/s11999-017-5394-6 28569372 PMC5539035

[47] Diep R, Garcia D. Does aspirin prevent venous thromboembolism? Hematology Am Soc Hematol Educ Program. 2020;2020(1):634-641. doi:10.1182/hematology.2020000150 33275727 PMC7727539

[48] Awtry EH, Loscalzo J. Aspirin. Circulation. 2000;101(10):1206-1218. doi:10.1161/01.CIR.101.10.1206 10715270

[49] Xing KH, Morrison G, Lim W, Douketis J, Odueyungbo A, Crowther M. Has the incidence of deep vein thrombosis in patients undergoing total hip/knee arthroplasty changed over time? a systematic review of randomized controlled trials. Thromb Res. 2008;123(1):24-34. doi:10.1016/j.thromres.2008.05.005 18620740

