# Supplementary material for: Venous Thromboembolism in Total Hip and Total Knee Arthroplasty
Source: JAMA Netw Open. 2023 Dec 1;6(12):e2345883. doi: 10.1001/jamanetworkopen.2023.45883 (PMC10692868; doi:10.1001/jamanetworkopen.2023.45883)
Supplement: Supplement 1. — eTable 1. International Classification of Diseases-10th Revision (ICD-10) Codes for Comorbidities eTable 2. National Drug Codes eTable 3. International Classification of Diseases-10th Revision (ICD-10) Codes for Venous Thromboembolism eTable 4. International Classification of Diseases-10th Revision (ICD-10) Codes for Bleed eTable 5. Chi-Squared Univariate Analysis of 30-Day Post-Operative Venous Thromboembolism (VTE) eTable 6. Multivariable Analysis of 90-Day Post-Operative Venous Thromboembolism and Bleeding eFigure 1. Box Plot of Propensity Scores for Receiving a Direct Oral Anticoagulant (DOAC) by DOAC and Aspirin Prior to Matching eTable 7. Overview of Patient and Surgical Variables of the Propensity Matched Cohort eFigure 2. Absolute Standardized Mean Differences Between Direct Oral Anticoagulant and Aspirin Patients [file jamanetwopen-e2345883-s001.pdf]

Supplemental Online Content

Simon SJ, Patell R, Zwicker JI, Kazi DS, Hollenbeck BL. Venous thromboembolism in total hip and total knee arthroplasty. *JAMA Netw Open.* 2023;6(12):e2345883. doi:10.1001/jamanetworkopen.2023.45883

eTable 1. International Classification of Diseases-10th Revision (ICD-10) Codes for Comorbidities
eTable 2. National Drug Codes
eTable 3. International Classification of Diseases-10th Revision (ICD-10) Codes for Venous Thromboembolism
eTable 4. International Classification of Diseases-10th Revision (ICD-10) Codes for Bleed
eTable 5. Chi-Squared Univariate Analysis of 30-Day Post-Operative Venous Thromboembolism (VTE)
eTable 6. Multivariable Analysis of 90-Day Post-Operative Venous Thromboembolism and Bleeding
eFigure 1. Box Plot of Propensity Scores for Receiving a Direct Oral Anticoagulant (DOAC) by DOAC and Aspirin Prior to Matching
eTable 7. Overview of Patient and Surgical Variables of the Propensity Matched Cohort
eFigure 2. Absolute Standardized Mean Differences Between Direct Oral Anticoagulant and Aspirin Patients

This supplemental material has been provided by the authors to give readers additional information about their work.

© 2023 Simon SJ et al. *JAMA Network Open.*

eTable 1. International Classification of Diseases-10th Revision (ICD-10) codes for comorbidities.

Comorbidity	ICD-10 code	Description
Obesity	E66.0	Obesity due to excess calories
	E66.01	Morbid (severe) obesity due to excess calories
	E66.09	Other obesity due to excess calories
	E66.1	Drug-induced obesity
	E66.2	Morbid (severe) obesity with alveolar hypoventilation
	E66.8	Other obesity
	E66.9	Obesity, unspecified
Chronic Kidney Disease	N18.2	Chronic kidney disease, stage 2 (mild)
	N18.3	Chronic kidney disease, stage 3 (moderate)
	N18.30	Chronic kidney disease, state 3 unspecified
	N18.31	Chronic kidney disease, stage 3a
	N18.32	Chronic kidney disease, stage 3b
	N18.4	Chronic kidney disease, stage 4 (severe)
	N18.5	Chronic kidney disease, stage 5
	N18.6	End stage renal disease
	N18.9	Chronic kidney disease, unspecified
Hereditary Hypercoagulable State	D68.51	factor V leiden mutation
	X83.2	FH: factor V leiden mutation
	X86.2	Thrombophilia, factor V Leiden mutation, remote, resolved
	D68.51	Activated protein C resistance
	D68.59	deficiency protein C/S
	D68.59	Hypercoagulable state including antithrombin, primary NEC, protein C and protein S deficiency
	D68.52	Prothrombin gene mutation
Antiphospholipid Antibody or Lupus Anticoagulant	D68.312	antiphospholipid antibody or lupus anticoagulant with hemorrhagic disorder
	D68.62	lupus anticoagulant
	D68.69	Secondary antiphospholipid antibody syndrome
	Z86.2	History of antiphospholipid antibody syndrome
	D68.61	antiphospholipid antibody
	R76.0	antiphospholipid antibody
History of Venous Thromboembolism	Z86.718	Personal history of other venous thrombosis and embolism
	see etable 3	Any venous thromboembolism or pulmonary embolism diagnosis prior to index surgery
Cancer	C02.x	Malignant neoplasm of other and unspecified parts of tongue
	C08.x	Malignant neoplasm of other and unspecified major salivary glands
	C09.x	Malignant neoplasm of tonsil
	C10.x	Malignant neoplasm of oropharynx
	C11.x	Malignant neoplasm of nasopharynx
	C13.x	Malignant neoplasm of hypopharynx
	C14.0	Malignant neoplasm of pharynx, unspecified
	C14.2	Waldeyer's ring

© 2023 Simon SJ et al. *JAMA Network Open.*

Comorbidity	ICD-10 code	Description
	C15.3	Malignant neoplasm of esophagus
**Comorbidity**	**ICD-10 code**	**Description**
Cancer	C16.x	Malignant neoplasm of stomach
	C17.x	Malignant neoplasm of small intestine
	C18.x	Malignant neoplasm of colon
	C21.x	Malignant neoplasm of anus and anal canal
	C22.x	Malignant neoplasm of liver and intrahepatic bile ducts
	C24.x	Malignant neoplasm of other and unspecified parts of biliary tract
	C25.x	Malignant neoplasm of pancreas
	C26.x	Malignant neoplasm of other and ill-defined digestive organs
	C31.x	Malignant neoplasm of accessory sinuses
	C32.x	Malignant neoplasm of larynx
	C34.x	Malignant neoplasm of bronchus and lung
	C38.x	Malignant neoplasm of heart, mediastinum and pleura
	C39.x	Malignant neoplasm of other and ill-defined sites in the respiratory system and intrathoracic organs
	C40.xx	Malignant neoplasm of bone and articular cartilage of limbs
	C41.xx	Malignant neoplasm of bone and articular cartilage of other and unspecified sites
	C45.x	Mesothelioma
	C46.x	Kaposi's sarcoma
	C48.x	Malignant neoplasm of retroperitoneum and peritoneum
	C49.xx	Malignant neoplasm of other connective and soft tissue
	C50.xx	Malignant neoplasm of breast
	C53.x	Malignant neoplasm of cervix uteri
	C54.x	Malignant neoplasm of corpus uteri
	C56.x	Malignant neoplasm of ovary
	C57.xx	Malignant neoplasm of female genital organ
	C62.xx	Malignant neoplasm of testis
	C63.xx	Malignant neoplasm of male genital organs
	C64.x	Malignant neoplasm of kidney, except renal pelvis
	C65.x	Malignant neoplasm of renal pelvis
	C66.x	Malignant neoplasm of ureter
	C67.x	Malignant neoplasm of bladder
	C68.x	Malignant neoplasm of urinary organ
	C70.x	Malignant neoplasm of meninges
	C71.x	Malignant neoplasm of brain
	C72.xx	Malignant neoplasm of spinal cord, cranial nerves and other parts of central nervous system
	C74.xx	Malignant neoplasm of adrenal gland
	C75.x	Malignant neoplasm of endocrine glands and related structures
	C76.xx	Malignant neoplasm of other specified ill-defined sites
	C77.x	Secondary and unspecified malignant neoplasm of lymph node
	C78.xx	Secondary malignant neoplasm of respiratory and digestive organs
	C79.xx	Secondary malignant neoplasm of unspecified site
	C80.x	Malignant neoplasm without specification of site
	C7A.xx	Malignant neuroendocrine tumors

© 2023 Simon SJ et al. *JAMA Network Open.*

eTable 2. National Drug Codes.

Medication	National Drug Codes
Warfarin	00056-0168, 00056-0169, 00056-0170, 00056-0172, 00056-0173, 00056-0174, 00056-0176, 00056-0188, 00056-0189, 00590-0324, 43353-0491, 43353-0492, 43353-0493, 43353-0494, 49999-0411, 50090-0028, 51138-0482, 51138-0483, 51138-0484, 51138-0485, 51138-0486, 51138-0487, 51138-0488, 51138-0489, 51138-0490, 52125-0053, 52125-0075, 52125-0079, 52125-0080, 52125-0091, 52125-0097, 52125-0099, 52125-0188, 52125-0189, 52125-0386, 52125-0387, 54569-0158, 54569-0159, 54569-4443, 54868-1259, 54868-2128, 54868-2129, 54868-2154, 54868-2252, 54868-2454, 54868-3399, 54868-4063, 54868-5255, 55154-7701, 55154-7702, 55154-7703, 55154-7704, 55154-7706, 55154-7707, 55154-7716, 55154-7717, 67544-0052, 67544-0070, 67544-0194, 67544-0195, 67544-0401, 69189-0675, 69189-0684, 00093-1712, 00093-1713, 00093-1714, 00093-1715, 00093-1716, 00093-1718, 00093-1719, 00093-1720, 00093-1721, 00093-1723, 00179-0191, 00179-0192, 00179-0193, 00179-0199, 00378-8801, 00378-8802, 00378-8803, 00378-8804, 00378-8805, 00378-8806, 00378-8810, 00378-8825, 00378-8875, 00555-0831, 00555-0832, 00555-0833, 00555-0834, 00555-0835, 00555-0869, 00555-0874, 00555-0925, 00555-0926, 16590-0340, 16590-0341, 17856-4034, 21695-0672, 21695-0673, 21695-0674, 21695-0675, 21695-0677, 21695-0801, 21695-0939, 21695-0940, 24236-0260, 24236-0353, 31722-0327, 31722-0328, 31722-0329, 31722-0330, 31722-0331, 31722-0332, 31722-0333, 31722-0334, 31722-0335, 33261-0355, 33261-0356, 33261-0357, 35356-0397, 35356-0571, 35356-0582, 35356-0906, 42549-0496, 42549-0497, 43063-0176, 43063-0218, 43063-0471, 43063-0655, 43353-0021, 43353-0023, 43353-0028, 43353-0029, 43353-0030, 43353-0033, 43353-0049, 43353-0050, 43353-0053, 43353-0054, 43353-0089, 43353-0142, 43353-0578, 43353-0579, 43353-0584, 43353-0587, 49349-0041, 49349-0140, 49349-0346, 49349-0542, 49349-0543, 49349-0555, 49349-0605, 49349-0618, 49349-0658, 49349-0716, 49349-0718, 49349-0796, 49349-0814, 49349-0824, 49349-0852, 49349-0861, 49349-0901, 49999-0923, 50090-0676, 50090-1009, 50090-1212, 50090-1213, 50090-1288, 50090-1289, 50090-1345, 50090-1361, 50090-1539, 50090-1756, 50090-1861, 50090-2032, 50090-2033, 50090-2034, 50090-2035, 50090-2036, 50090-2037, 50090-2038, 50090-2039, 50090-2141, 50090-2582, 50090-2586, 50090-2613, 50090-2617, 50090-2645, 50090-2646, 50090-2654, 50090-2936, 50090-3110, 50090-3120, 50090-3263, 50090-5004, 50090-5058, 50090-5390, 50090-5439, 50090-5440, 50090-5464, 50090-5465, 50436-6357, 50436-6358, 50436-6360, 50436-6363, 50436-6364, 50436-9933, 50436-9946, 51138-0054, 51138-0055, 51138-0056, 51138-0057, 51138-0058, 51138-0059, 51138-0060, 51138-0061, 51138-0062, 51407-0341, 51407-0342, 51407-0343, 51407-0344, 51407-0345, 51407-0346, 51407-0347, 51407-0348, 51407-0349, 51407-0784, 51407-0785, 51407-0786, 51407-0787, 51407-0788, 51407-0789, 51407-0790, 51407-0791, 51407-0792, 51655-0278, 51655-0282, 51655-0283, 51672-4027, 51672-4028, 51672-4029, 51672-4030, 51672-4031, 51672-4032, 51672-4033, 51672-4034, 51672-4035, 52125-0385, 52125-0432, 52125-0501, 52125-0629, 52125-0906, 52125-0909, 52125-0910, 53217-0001, 53217-0018, 53217-0219, 53217-0235, 53808-0334, 53808-0335, 53808-0970, 53808-0985, 53808-0989, 53808-0994, 53808-0997, 54569-4934, 54569-5868, 54569-5869, 54569-6225, 54868-4286, 54868-4349, 54868-4400, 54868-4402, 54868-4422, 54868-4871, 54868-4873, 54868-4950, 54868-5258, 55154-0876, 55154-0877, 55154-0878, 55154-0879, 55154-0880, 55154-0883, 55154-0884, 55154-4695, 55154-4696, 55154-4697, 55289-0340, 55289-0773, 55700-0005, 57237-0119, 57237-0120, 57237-0121, 57237-0122, 57237-0123, 57237-0124, 57237-0125, 57237-0126, 57237-0127, 58118-0033, 58118-0041, 58118-0060, 58118-0063, 58118-0064, 58118-1715, 58118-4027, 58118-4028, 58118-4029, 58118-4030, 58118-4031,

© 2023 Simon SJ et al. *JAMA Network Open.*

Medication	National Drug Codes
Warfarin	58118-4032, 58118-4033, 58118-4034, 58118-4035, 58517-0360, 60429-0784, 60429-0785, 60429-0786, 60429-0787, 60429-0788, 60429-0789, 60429-0790, 60429-0791, 60429-0792, 60760-0040, 60760-0041, 60760-0043, 60760-0124, 60760-0706, 61786-0342, 61786-0553, 61786-0569, 61786-0570, 61786-0571, 61786-0588, 61786-0593, 61786-0615, 61786-0626, 61919-0341, 62584-0984, 62584-0994, 63187-0674, 63187-0745, 63187-0750, 63629-2548, 63629-3177, 63629-4017, 63629-4122, 63629-4417, 63629-4543, 63629-4748, 63629-5257, 63629-6342, 63629-6400, 63629-6401, 63629-6977, 63629-8146, 63629-8147, 63629-8843, 64205-0032, 64725-0056, 64725-0833, 65162-0761, 65162-0762, 65162-0763, 65162-0764, 65162-0765, 65162-0766, 65162-0767, 65162-0768, 65162-0769, 65841-0052, 65841-0053, 65841-0054, 65841-0055, 65841-0056, 65841-0057, 65841-0058, 65841-0059, 65841-0064, 66267-0268, 66267-0277, 66267-0285, 66267-0672, 67296-1284, 67296-1285, 67544-0318, 68071-3018, 68071-3073, 68071-4384, 68071-4385, 68071-4479, 68071-4725, 68071-4772, 68071-5003, 68071-5004, 68084-0027, 68382-0052, 68382-0053, 68382-0054, 68382-0055, 68382-0056, 68382-0057, 68382-0058, 68382-0059, 68382-0064, 68788-7357, 68788-7358, 68788-7515, 70518-0253, 70518-0255, 70518-0260, 70518-0261, 70518-0266, 70518-0273, 70518-0279, 70518-0280, 70518-0299, 70518-0727, 70518-1006, 70518-1467, 70518-1492, 70518-2380, 70518-2381, 70518-2382, 70518-2383, 70518-2394, 70518-2395, 70518-2717, 70518-2816, 70518-2900, 70786-1712, 70786-1713, 70786-1714, 70786-1715, 70786-1716, 70786-1718, 70786-1721, 70934-0585, 70934-0586, 70934-0587, 70934-0588, 70934-0589, 70934-0820, 70934-0821, 71335-0243, 71335-0270, 71335-0348, 71335-0452, 71335-0580, 71335-0637, 71335-0840, 71335-1059, 71335-1595, 71335-1615, 71335-1718, 71335-1786, 71610-0173, 71610-0179, 71610-0448, 71610-0458, 71610-0462, 71610-0480, 71610-0490, 71610-0491, 71610-0498, 71610-0519, 71610-0574, 71610-0575, 71610-0589, 71610-0593, 72189-0210, 76282-0327, 76282-0328, 76282-0329, 76282-0330, 76282-0331, 76282-0332, 76282-0333, 76282-0334, 76282-0335
Enoxaparin	00548-5601, 00548-5602, 00548-5603, 00548-5604, 00548-5605, 00548-5606, 00548-5607, 00548-5608, 00548-5631, 00548-5632, 00548-5633, 00548-5634, 00548-5635, 00548-5636, 00548-5637, 00703-8510, 00703-8530, 00703-8540, 00703-8560, 00703-8580, 00703-8610, 00703-8680, 00781-3119, 00781-3121, 00781-3122, 00781-3133, 00781-3224, 00781-3238, 00781-3246, 00781-3256, 00781-3262, 00781-3268, 00781-3298, 00781-3299, 00781-3356, 00781-3428, 00781-3500, 00781-3612, 00781-3655, 00955-1003, 00955-1004, 00955-1006, 00955-1008, 00955-1010, 00955-1012, 00955-1015, 00955-1016, 11797-0757, 11797-0758, 11797-0759, 11797-0760, 11797-0761, 11797-0762, 11797-0763, 16714-0006, 16714-0016, 16714-0026, 16714-0036, 16714-0046, 16714-0056, 16714-0066, 50090-3445, 55154-5433, 55154-5434, 55154-5435, 55154-5436, 55154-5437, 55154-5440, 55154-5441, 55154-5442, 55154-5443, 55154-5444, 55154-6688, 55154-6689, 55154-6690, 55154-6691, 55154-6692, 55154-9399, 55154-9576, 55154-9578, 55154-9579, 55154-9580, 60505-0791, 60505-0792, 60505-0793, 60505-0794, 60505-0795, 60505-0796, 60505-0798, 62037-0839, 62037-0849, 62037-0861, 62037-0862, 62037-0863, 62037-0864, 62037-0866, 63323-0531, 63323-0533, 63323-0535, 63323-0537, 63323-0539, 63323-0559, 63323-0564, 63323-0565, 63323-0566, 63323-0568, 63323-0569, 63323-0584, 63323-0586, 63323-0589, 63323-0605, 63323-0607, 63323-0609, 63323-0655, 68001-0457, 68001-0458, 68001-0459, 68001-0460, 68001-0461, 68001-0462, 68001-0463, 68001-0464, 70710-1757, 70710-1758, 70710-1759, 70710-1760, 70710-1761, 70710-1762, 70710-1763, 71288-0410, 71288-0411, 76420-0086
Rivaroxaban	50090-3625, 50090-3639, 50090-4468, 50090-4469, 50458-0577, 50458-0578, 50458-0579, 50458-0580, 50458-0584, 55154-1422, 55154-1423, 55154-1424, 69189-0578

© 2023 Simon SJ et al. *JAMA Network Open.*

Medication	National Drug Codes
Apixaban	14445-0149, 14445-0150, 00003-0893, 00003-0894, 00003-3764, 50090-1436, 50090-1437, 55154-0612, 55154-0613, 63629-7747, 67296-1673, 70518-1861
Aspirin	00498-0113, 67060-0232, 12634-0120, 00067-0149, 00113-0416, 00113-0773, 00113-1411, 00113-1919, 00185-0724, 00280-2000, 00280-2110, 00280-2605, 00363-0157, 00363-0183, 00363-0227, 00363-0691, 00378-6117, 00404-0164, 00404-0165, 00404-0710, 00404-0712, 00498-0114, 00536-1053, 00536-1054, 00536-1148, 00536-1232, 00536-1284, 00536-3305, 00536-3313, 00591-3546, 00591-3551, 00603-0168, 00603-0169, 00615-0522, 00615-0564, 00615-8280, 00615-8324, 00904-2009, 00904-2011, 00904-2013, 00904-2015, 00904-2019, 00904-6712, 00904-6744, 00904-6784, 00904-6809, 00904-6811, 00924-0104, 00924-0105, 00924-0106, 00924-0204, 10202-0416, 11673-0371, 11673-0378, 11673-0416, 11788-0302, 11822-0157, 11822-0183, 11822-2270, 11868-0157, 12634-0438, 15127-0227, 15127-0738, 16103-0357, 16103-0365, 17714-0010, 17714-0011, 17856-0901, 17856-0922, 21130-0081, 21130-0157, 21130-0167, 21130-0957, 21695-0570, 21695-0666, 23155-0145, 24236-0734, 24385-0416, 24385-0429, 27854-0105, 30142-0370, 30142-0391, 30142-0416, 30142-0627, 33261-0153, 33992-0157, 33992-0249, 35418-0232, 36800-0127, 36800-0157, 36800-0983, 37205-0145, 37205-0429, 37205-0668, 37808-0031, 37808-0157, 37835-0738, 41163-0157, 41163-0370, 41163-0405, 41163-0416, 41163-0522, 41190-0416, 41226-0411, 41250-0118, 41250-0371, 41250-0416, 41250-0681, 41268-0416, 41520-0157, 41520-0370, 41520-0416, 41520-0651, 42254-0141, 42507-0370, 42681-0013, 42681-7075, 42961-0044, 42961-0113, 46122-0292, 46122-0596, 46122-0635, 46122-0691, 46994-0157, 47682-0011, 47682-0085, 47682-0095, 47682-0097, 47682-0098, 47682-0099, 47682-0104, 47682-0116, 47682-0119, 47682-0151, 47682-0456, 47682-0616, 47682-0617, 47682-0618, 47682-0620, 47682-0621, 47682-0622, 47682-0805, 47682-0806, 47682-0905, 47682-0906, 48083-0654, 48433-0305, 49035-0157, 49035-0355, 49035-0370, 49035-0416, 49035-0722, 49314-1163, 49314-1173, 49348-0001, 49348-0937, 49349-0163, 49349-0386, 49483-0011, 49483-0052, 49738-0416, 49738-0781, 49781-0095, 49781-0096, 50090-0002, 50090-0003, 50090-0004, 50090-0103, 50090-1617, 50090-2389, 50090-3450, 50090-4462, 50090-4858, 50269-0005, 50269-0006, 50332-0102, 50564-0490, 50804-0370, 50844-0157, 50844-0227, 50844-0249, 50844-0254, 50844-0957, 51532-0232, 51596-0001, 51596-0006, 51645-0716, 52124-0011, 52204-0111, 52682-0023, 52959-0018, 53185-0249, 53808-1028, 53808-1034, 53943-0157, 54162-0940, 54569-0005, 54569-0014, 54569-0337, 54738-0111, 54738-0541, 54868-1198, 54868-2094, 54868-2405, 55154-6755, 55154-6808, 55154-6828, 55301-0157, 55301-0227, 55315-0157, 55316-0416, 55319-0492, 55670-0131, 55670-0616, 55670-0620, 55670-0676, 55700-0338, 55758-0312, 55910-0157, 55910-0249, 56062-0118, 56062-0416, 57243-0157, 57344-0018, 57344-0081, 57896-0109, 57896-0901, 57896-0904, 57896-0921, 57896-0922, 58118-2013, 59088-0120, 59726-0370, 59779-0137, 59779-0157, 59779-0183, 59779-0227, 59779-0249, 59779-0389, 59779-0416, 59779-0733, 59779-0772, 59961-0139, 59961-0616, 59961-0620, 60683-0106, 60760-0047, 60760-0161, 60760-0718, 60760-0854, 60760-0891, 60760-0921, 61010-4407, 61245-0116, 61245-0152, 61245-0616, 61245-0620, 62011-0020, 62011-0040, 62011-0405, 62011-0432, 62211-0225, 62211-0312, 62211-0839, 62732-8006, 62732-8009, 63187-0530, 63629-1762, 63868-0352, 63868-0898, 63940-0320, 63940-0321, 63941-0157, 63941-0249, 63941-0370, 63981-0227, 64092-0110, 64092-0141, 64980-0175, 65437-0038, 65923-0249, 65923-0507, 66267-0026, 66715-6704, 66715-6840, 67046-0162, 67046-0164, 67046-0169, 67046-0170, 67091-0110, 67510-0008, 67544-0468, 67751-0009, 67751-0172, 68016-0045, 68016-0722, 68016-0747, 68071-3189, 68071-4081, 68071-4667, 68071-5192, 68084-0848, 68196-0157, 68210-0025, 68210-2500, 68210-4093, 68308-0845, 68391-0416, 68788-0678, 68788-7077, 68788-7582, 68788-7794, 68788-9134, 68998-0018, 68998-0081, 69103-2501, 69168-0312, 69168-0331,

© 2023 Simon SJ et al. *JAMA Network Open.*

Medication	National Drug Codes
Aspirin	69168-0400, 69189-0921, 69189-2015, 69517-0115, 69618-0015, 69618-0016, 69790-0098, 69790-0616, 69790-0620, 69842-0927, 70000-0014, 70000-0035, 70000-0107, 70000-0147, 70000-0253, 70000-0359, 70000-0507, 70000-0564, 70262-0302, 70264-0013, 70385-2005, 70518-0030, 70518-1059, 70518-1446, 70518-1949, 70518-2684, 70518-3069, 70677-0071, 70677-0092, 70934-0561, 71141-0117, 71205-0318, 71205-0517, 71335-0157, 71335-0513, 71335-0568, 71476-0104, 71610-0034, 71679-0050, 72036-0018, 72036-0181, 72189-0187, 72288-0416, 73057-0357, 73057-0365, 73097-0003, 73408-0099, 73408-0616, 73408-0620, 75826-0104, 76000-0370, 76237-0112, 76413-0303, 80136-0901, 81238-0232, 49483-0331, 50090-2957, 55154-1463, 63739-0523, 70000-0237, 00440-1130, 00067-0143, 00280-2020, 00280-2610, 79739-7182, 49483-0382, 00574-7036, 00067-6424, 00113-0259, 00113-0274, 00113-0277, 00113-0467, 00113-0535, 00113-7467, 00182-1061, 00280-2080, 00280-2090, 00363-0218, 00363-0397, 00363-0414, 00363-0439, 00363-0563, 00363-0587, 00363-6450, 00440-7135, 00536-1004, 00536-1008, 00536-1149, 00536-1234, 00536-3086, 00536-3297, 00603-0024, 00603-0026, 00615-3508, 00615-3568, 00615-7863, 00615-8058, 00615-8238, 00615-8257, 00615-8323, 00904-4040, 00904-6288, 00904-6713, 00904-6751, 00904-6783, 00904-6794, 10056-0535, 10135-0173, 10135-0689, 10202-0259, 11534-0073, 11534-0155, 11673-0027, 11673-0181, 11673-0277, 11673-0467, 11673-0563, 11673-0702, 11788-0301, 11822-0218, 11822-0397, 11822-0439, 11822-0600, 11822-0899, 11822-2180, 11822-6451, 15127-0228, 15127-0241, 15127-0600, 15127-0874, 16103-0351, 16103-0356, 16103-0366, 17714-0009, 17714-0121, 17856-3086, 17856-6794, 21130-0218, 21130-0397, 21130-0414, 21130-0563, 21130-0600, 21130-0645, 21130-0918, 21695-0684, 24385-0028, 24385-0278, 24385-0364, 24385-0535, 24385-0541, 30142-0027, 30142-0103, 30142-0104, 30142-0274, 30142-0440, 30142-0467, 30142-0699, 33261-0152, 33992-0218, 33992-2270, 33992-2883, 35356-0900, 36800-0027, 36800-0030, 36800-0146, 36800-0259, 36800-0274, 36800-0277, 36800-0467, 36800-0545, 36800-0880, 36800-0968, 37012-0826, 37205-0369, 37205-0467, 37205-0510, 37205-0708, 37205-0757, 37808-0246, 37808-0274, 37808-0277, 37808-0467, 37808-0535, 37808-0600, 37808-0627, 37808-0630, 37808-0867, 41163-0218, 41163-0274, 41163-0397, 41163-0414, 41163-0467, 41163-0563, 41163-0797, 41163-0918, 41163-0945, 41190-0467, 41226-0481, 41250-0146, 41250-0274, 41250-0277, 41250-0467, 41250-0535, 41250-0600, 41250-0780, 41268-0535, 41520-0030, 41520-0103, 41520-0228, 41520-0259, 41520-0274, 41520-0440, 41520-0467, 42507-0027, 42507-0274, 42507-0467, 42507-0535, 43063-0862, 46122-0180, 46122-0182, 46122-0262, 46122-0598, 46122-0615, 46994-0600, 48433-0303, 49035-0274, 49035-0467, 49035-0563, 49035-0914, 49348-0191, 49348-0498, 49348-0653, 49348-0756, 49348-0757, 49348-0980, 49348-0981, 49349-0868, 49483-0328, 49483-0334, 49483-0381, 49483-0387, 49483-0390, 49483-0481, 49614-0467, 49614-0535, 49638-0001, 49638-0101, 49638-0102, 49638-0115, 49638-0481, 49738-0023, 49738-0077, 49738-0274, 49738-0277, 49738-0317, 49738-0467, 49738-0784, 49738-0786, 49781-0084, 49781-0094, 49781-0097, 50090-0552, 50090-0909, 50090-2832, 50090-2898, 50090-2940, 50090-4830, 50090-4832, 50090-5476, 50090-5533, 50436-0127, 50804-0227, 50804-0880, 50844-0218, 50844-0219, 50844-0563, 50844-0600, 51645-0717, 51824-0055, 51824-0057, 51824-0077, 51824-0164, 51824-0547, 52124-0012, 52204-0103, 53002-1673, 53329-0020, 53329-0985, 53943-0080, 54257-0274, 54569-4233, 54738-0128, 54738-0559, 54868-2440, 54868-4864, 55154-0748, 55154-1473, 55154-1476, 55154-1493, 55154-1494, 55154-6752, 55154-8146, 55301-0218, 55301-0414, 55301-0600, 55301-0821, 55312-0077, 55312-0103, 55312-0104, 55312-0259, 55312-0277, 55312-0440, 55312-0467, 55312-0535, 55315-0103, 55315-0104, 55315-0218, 55315-0227, 55315-0397, 55315-0600, 55315-0645, 55315-0867, 55316-0227, 55316-0274,

© 2023 Simon SJ et al. *JAMA Network Open.*

Medication	National Drug Codes
Aspirin	55316-0467, 55316-0535, 55319-0218, 55319-0227, 55319-0467, 55319-0600, 55319-0601, 55700-0746, 55700-0842, 55758-0005, 55910-0274, 55910-0414, 55910-0438, 55910-0467, 55910-0563, 55910-0600, 55910-0945, 56062-0027, 56062-0259, 56062-0274, 56062-0440, 56062-0467, 56062-0535, 56062-0719, 56062-0867, 57344-0080, 57344-0105, 57344-0146, 57344-0152, 57896-0911, 57896-0981, 57896-0985, 58118-0387, 59726-0021, 59726-0065, 59726-0227, 59726-0440, 59726-0867, 59779-0218, 59779-0274, 59779-0414, 59779-0439, 59779-0467, 59779-0600, 59779-0605, 59779-0727, 59779-0945, 60577-0005, 60760-0045, 61715-0040, 61786-0335, 61786-0532, 61786-0685, 61786-0807, 61919-0420, 62011-0003, 62011-0019, 62011-0021, 62011-0028, 62011-0212, 62011-0404, 62011-0480, 62049-0001, 62107-0026, 62107-0027, 62211-0220, 62211-0838, 62309-0777, 62309-0779, 62732-8005, 62732-8008, 63187-0297, 63187-0717, 63187-0882, 63548-0046, 63548-0227, 63629-2825, 63629-6326, 63629-6356, 63629-7719, 63739-0212, 63739-0434, 63739-0522, 63868-0029, 63868-0240, 63868-0332, 63868-0354, 63868-0363, 63868-0373, 63868-0469, 63940-0218, 63940-0333, 63940-0609, 63941-0081, 63941-0103, 63941-0227, 63941-0397, 63941-0440, 63941-0441, 63941-0600, 63941-0NEW, 63981-0563, 64092-0119, 64092-0150, 64092-0199, 64092-0218, 64725-0981, 65084-0555, 65155-0981, 67046-0163, 67046-0165, 67046-0166, 67046-0167, 67046-0168, 67091-0122, 67091-0123, 67091-0124, 67091-0389, 67544-0524, 68016-0089, 68016-0254, 68016-0642, 68016-0689, 68016-0690, 68071-3101, 68071-4148, 68071-4200, 68071-4254, 68071-4449, 68071-4762, 68151-2904, 68163-0081, 68196-0600, 68196-0900, 68196-0901, 68210-0015, 68210-0150, 68210-0220, 68210-4098, 68210-4128, 68210-5100, 68391-0063, 68391-0227, 68391-0867, 68788-0186, 68788-7107, 68788-7792, 68788-7862, 68788-8925, 68788-9671, 68998-0080, 68998-0105, 68998-0146, 69168-0288, 69168-0318, 69168-0372, 69189-4040, 69517-0136, 69536-0181, 69536-0281, 69607-0867, 69618-0014, 69618-0017, 69618-0046, 69618-0066, 69842-0826, 70000-0102, 70000-0103, 70000-0105, 70000-0170, 70000-0178, 70000-0202, 70000-0203, 70000-0218, 70000-0419, 70000-0420, 70000-0428, 70253-0218, 70253-0397, 70253-0600, 70297-0018, 70518-0699, 70518-0838, 70518-0923, 70518-0928, 70518-1507, 70518-1649, 70518-1982, 70518-2069, 70518-2171, 70518-2422, 70518-2596, 70518-2733, 70518-3025, 70677-0070, 70677-0132, 70677-0136, 70692-0126, 70692-0158, 70692-0161, 70934-0495, 71141-0005, 71141-0158, 71205-0112, 71205-0328, 71205-0442, 71335-0195, 71335-0340, 71335-0608, 71335-0622, 71335-0932, 71335-1382, 71399-8627, 71406-0128, 71476-0101, 71476-0102, 71476-0103, 71679-0010, 71872-7087, 71872-7154, 72036-0080, 72036-0105, 72036-0146, 72036-0152, 72036-0205, 72036-0252, 72090-0001, 72189-0188, 72189-0274, 72288-0467, 72476-0105, 72476-0152, 72476-0205, 72476-0252, 72476-0867, 72615-0064, 72629-0020, 72714-0351, 72789-0039, 73057-0356, 73057-0366, 75942-0001, 76000-0179, 76000-0180, 76000-0231, 76000-0414, 76168-0009, 76237-0113, 76413-0330, 76413-0355, 49483-0349, 55505-0168, 42787-0101

© 2023 Simon SJ et al. *JAMA Network Open.*

eTable 3. International Classification of Diseases-10th Revision (ICD-10) codes for venous thromboembolism.

ICD-10 Code	Description
I80.1x	Phlebitis and thrombophlebitis of femoral vein
I80.20x	Phlebitis and thrombophlebitis of unspecified deep vessels of lower extremities
I80.21x	Phlebitis and thrombophlebitis of iliac vein
I80.22x	Phlebitis and thrombophlebitis of popliteal vein
I80.29x	Phlebitis and thrombophlebitis of other deep vessels of lower extremities
I82.40x	Acute embolism and thrombosis of unspecified deep veins of lower extremity
I82.41x	Acute embolism and thrombosis of femoral vein
I82.42x	Acute embolism and thrombosis of iliac vein
I82.43x	Acute embolism and thrombosis of popliteal vein
I82.44x	Acute embolism and thrombosis of tibial vein
I82.49x	Acute embolism and thrombosis of other specified deep vein of lower extremity
I82.4Yx	Acute embolism and thrombosis of unspecified deep veins of proximal lower extremity
I82.4Zx	Acute embolism and thrombosis of unspecified deep veins of distal lower extremity
I82.890	Acute embolism and thrombosis of other specified veins
I82.90	Acute embolism and thrombosis of unspecified vein
T84.86XA	Thrombosis due to internal orthopedic prosthetic devices, implants and grafts, initial encounter
T84.81XA	Embolism due to internal orthopedic prosthetic devices, implants and grafts, initial encounter
I26.02	Saddle embolus of pulmonary artery with acute cor pulmonale
I26.09	Other pulmonary embolism with acute cor pulmonale
I26.92	Saddle embolus of pulmonary artery without acute cor pulmonale
I26.93	Single subsegmental pulmonary embolism without acute cor pulmonale
I26.94	Multiple subsegmental pulmonary emboli without acute cor pulmonale
I26.99	Other pulmonary embolism without acute cor pulmonale

© 2023 Simon SJ et al. *JAMA Network Open.*

eTable 4. International Classification of Diseases-10th Revision (ICD-10) codes for bleed.

ICD-10 code	Description
H05.23x	Hemorrhage of orbit
H21.0x	Hyphema
H31.3xx	Unspecified choroidal hemorrhage
H31.31x	Expulsive choroidal hemorrhage
H31.41x	Hemorrhagic choroidal detachment
H35.6x	Retinal hemorrhage
H35.73x	Hemorrhagic detachment of retinal pigment epithelium
H43.1x	Vitreous hemorrhage
H44.81x	Hemophthalmos
H47.02x	Hemorrhage in optic nerve sheath
H61.12x	Hematoma of pinna
I23.0	Hemopericardium as current complication following acute myocardial infarction
I31.2	Hemopericardium
I60.xx	Nontraumatic subarachnoid hemorrhage
I61.x	Nontraumatic intracerebral hemorrhage
I62.0x	Nontraumatic subdural hemorrhage
I62.1	Nontraumatic extradural hemorrhage
I62.9	Nontraumatic intracranial hemorrhage, unspecified
I85.01	Esophageal varices with bleeding
K22.11	Ulcer of esophagus with bleeding
K25.0	Acute gastric ulcer with hemorrhage
K25.2	Acute gastric ulcer with both hemorrhage and perforation
K25.4	Chronic or unspecified gastric ulcer with hemorrhage
K25.6	Chronic or unspecified gastric ulcer with both hemorrhage and perforation
K26.0	Acute duodenal ulcer with hemorrhage
K26.2	Acute duodenal ulcer with both hemorrhage and perforation
K26.4	Chronic or unspecified duodenal ulcer with hemorrhage
K26.6	Chronic or unspecified duodenal ulcer with both hemorrhage and perforation
K27.0	Acute peptic ulcer, site unspecified, with hemorrhage
K27.2	Acute peptic ulcer, site unspecified, with both hemorrhage and perforation
K27.4	Chronic or unspecified peptic ulcer, site unspecified, with hemorrhage
K27.6	Chronic or unspecified peptic ulcer, site unspecified, with both hemorrhage and perforation
K28.0	Acute gastrojejunal ulcer with hemorrhage
K28.2	Acute gastrojejunal ulcer with both hemorrhage and perforation
K28.4	Chronic or unspecified gastrojejunal ulcer with hemorrhage
K28.6	Chronic or unspecified gastrojejunal ulcer with both hemorrhage and perforation
K29.01	Acute gastritis with bleeding
K29.21	Alcoholic gastritis with bleeding
K29.31	Chronic superficial gastritis with bleeding
K29.41	Chronic atrophic gastritis with bleeding
K29.51	Unspecified chronic gastritis with bleeding
K29.61	Other gastritis with bleeding

© 2023 Simon SJ et al. *JAMA Network Open.*

ICD-10 code	Description
K29.71	Gastritis, unspecified, with bleeding
K29.81	Duodenitis with bleeding
K29.91	Gastroduodenitis, unspecified, with bleeding
K31.811	Angiodysplasia of stomach and duodenum with bleeding
K50.011	Crohn's disease of small intestine with rectal bleeding
K50.111	Crohn's disease of large intestine with rectal bleeding
K50.811	Crohn's disease of both small and large intestine with rectal bleeding
K50.911	Crohn's disease, unspecified, with rectal bleeding
K51.011	Ulcerative (chronic) pancolitis with rectal bleeding
K51.211	Ulcerative (chronic) proctitis with rectal bleeding
K51.311	Ulcerative (chronic) rectosigmoiditis with rectal bleeding
K51.411	Inflammatory polyps of colon with rectal bleeding
K51.511	Left sided colitis with rectal bleeding
K51.811	Other ulcerative colitis with rectal bleeding
K51.911	Ulcerative colitis, unspecified with rectal bleeding
K55.21	Angiodysplasia of colon with hemorrhage
K57.01	Diverticulitis of small intestine with perforation and abscess with bleeding
K57.11	Diverticulosis of small intestine without perforation or abscess with bleeding
K57.13	Diverticulitis of small intestine without perforation or abscess with bleeding
K57.21	Diverticulitis of large intestine with perforation and abscess with bleeding
K57.31	Diverticulosis of large intestine without perforation or abscess with bleeding
K57.33	Diverticulitis of large intestine without perforation or abscess with bleeding
K57.41	Diverticulitis of both small and large intestine with perforation and abscess with bleeding
K57.51	Diverticulosis of both small and large intestine without perforation or abscess with bleeding
K57.53	Diverticulitis of both small and large intestine without perforation or abscess with bleeding
K57.81	Diverticulitis of intestine, part unspecified, with perforation and abscess with bleeding
K57.91	Diverticulosis of intestine, part unspecified, without perforation or abscess with bleeding
K57.93	Diverticulitis of intestine, part unspecified, without perforation or abscess with bleeding
K62.5	Hemorrhage of anus and rectum
K66.1	Hemoperitoneum
K92.0	Hematemesis
K92.1	Melena
K92.2	Gastrointestinal hemorrhage, unspecified
L76.32	Postprocedural hematoma of skin and subcutaneous tissue following other procedure
L76.34	Postprocedural hematoma of skin and subcutaneous tissue following other procedure
M25.0xx	Hemarthrosis
M79.81	Nontraumatic hematoma of soft tissue
N83.7	Hematoma of broad ligament
N89.7	Hematocolpos
N92.0	Excessive and frequent menstruation with regular cycle
N92.1	Excessive and frequent menstruation with irregular cycle

© 2023 Simon SJ et al. *JAMA Network Open.*

ICD-10 code	Description
N92.5	Other specified irregular menstruation
N93.8	Other specified abnormal uterine and vaginal bleeding
N93.9	Abnormal uterine and vaginal bleeding, unspecified
N99.820	Postprocedural hemorrhage of a genitourinary system organ or structure following a genitourinary system procedure
O71.7	Obstetric hematoma of pelvis
R04.0	Epistaxis
R04.1	Hemorrhage from throat
R04.2	Hemoptysis
R04.89	Hemorrhage from other sites in respiratory passages
R04.9	Hemorrhage from respiratory passages, unspecified
R31.0	Gross hematuria
R31.9	Hematuria, unspecified
R58	Hemorrhage, not elsewhere classified
S06.34xx	Traumatic hemorrhage of right cerebrum
S06.35xx	Traumatic hemorrhage of left cerebrum
S06.36xx	Traumatic hemorrhage of cerebrum, unspecified
S06.360A	Traumatic hemorrhage of cerebrum, unspecified, without loss of consciousness, initial encounter
S06.37xx	Contusion, laceration, and hemorrhage of cerebellum
S06.38xx	Contusion, laceration, and hemorrhage of brainstem
S06.5Xxx	Traumatic subdural hemorrhage
S06.6Xxx	Traumatic subarachnoid hemorrhage
S06.6X9A	Traumatic subarachnoid hemorrhage with loss of consciousness of unspecified duration, initial encounter
T14.8XXA	Hematoma
T85.838A	Hemorrhage associated with implant

© 2023 Simon SJ et al. *JAMA Network Open.*

eTable 5. Chi-squared univariate analysis of 30-day post-operative venous thromboembolism (VTE).

**Variables**	**No VTE**<br>No. (%)	**VTE**<br>No. (%)	P-Value
Total	28914 (98.8)	350 (1.2)	
Age: < 65	23684 (98.9)	268 (1.1)	**0.01**
Age: ≥ 65	5230 (98.5)	82 (1.5)	
Male	12060 (98.7)	164 (1.3)	0.05
Female	16854 (98.9)	186 (1.1)	
History of Venous Thromboembolism	679 (92.9)	52 (7.1)	**<.001**
No History of Venous Thromboembolism	28235 (99.0)	298 (1.0)	
Obesity	8122 (98.9)	90 (1.1)	0.32
No Obesity	20792 (98.8)	260 (1.2)	
Cancer	1303 (98.1)	25 (1.9)	**0.02**
No Cancer	27611 (98.8)	325 (1.2)	
Chronic Kidney Disease	1118 (97.9)	24 (2.1)	**0.004**
No Chronic Kidney Disease	27796 (98.8)	326 (1.2)	
Antiphospholipid Antibody or Lupus Anticoagulant	247 (96.9)	8 (3.1)	**0.004**
No Antiphospholipid Antibody or Lupus Anticoagulant	28667 (98.8)	342 (1.2)	
Hereditary Hypercoagulable Diagnosis	139 (92.7)	11 (7.3)	**<.001**
No Hereditary Hypercoagulable Diagnosis	28775 (98.8)	339 (1.2)	
Hip Arthroplasty	10853 (99.1)	95 (0.9)	**<.001**
Knee Arthroplasty	18061 (98.6)	255 (1.4)	
Outpatient-Hospital-Based	1236 (99.1)	11 (0.9)	
Inpatient	27559 (98.8)	338 (1.2)	0.54
Ambulatory Surgical Center	119 (99.2)	1 (0.8)	
Surgery Year: 2017	11203 (98.7)	147 (1.3)	
Surgery Year: 2018	11345 (98.8)	136 (1.2)	0.32
Surgery Year: 2019	6366 (99.0)	67 (1.0)	
Length of Stay: 0 Days	4673 (98.8)	58 (1.2)	
Length of Stay: 1-2 Days	9228 (99.0)	90 (1.0)	**0.03**
Length of Stay: 3-4 Days	14209 (98.7)	186 (1.3)	
Length of Stay: ≥ 5 Days	804 (98.1)	16 (1.9)	
Thromboprophylactic Medication			
Aspirin	10009 (99.3)	73 (0.7)	
Apixaban	3215 (98.8)	38 (1.2)	
Rivaroxaban	6989 (98.9)	79 (1.1)	**<.001**
Enoxaparin	5664 (98.3)	100 (1.7)	
Warfarin	3037 (98.1)	60 (1.9)	

© 2023 Simon SJ et al. *JAMA Network Open.*

eTable 6. Multivariable analysis of 90-day post-operative venous thromboembolism and bleeding.

Variables	Venous Thromboembolism		Bleed	
	Odds Ratio (95% CI)	P-value	Odds Ratio (95% CI)	P-value
Age: ≥ 65 vs < 65	1.06 (0.86-1.31)	0.59	1.28 (1.13-1.45)	<.001
Male vs Female	1.33 (1.12-1.58)	0.001	0.91 (0.82-1.01)	0.08
Length of Stay: 1-2 days vs 0 days	0.97 (0.74-1.28)	0.82	1.02 (0.86-1.21)	0.81
Length of Stay: 3-4 days vs 0 days	1.13 (0.88-1.44)	0.35	1.30 (1.12-1.52)	0.001
Length of Stay: ≥ 5 days vs 0 days	1.37 (0.84-2.22)	0.21	1.90 (1.44-2.51)	<.001
Knee vs Hip Arthroplasty	1.46 (1.20-1.76)	<.001	1.08 (0.97-1.20)	0.17
Obesity	0.88 (0.72-1.07)	0.19	1.03 (0.92-1.15)	0.62
Cancer	1.35 (0.95-1.92)	0.10	1.34 (1.09-1.66)	0.007
Chronic Kidney Disease	1.47 (1.03-2.10)	0.03	1.28 (1.02-1.61)	0.03
History of VTE	8.18 (6.39-10.47)	<.001	1.24 (0.93-1.66)	0.14
ALPA or Lupus Anticoagulant	1.32 (0.66-2.65)	0.43	1.94 (1.29-2.91)	0.001
Hereditary Hypercoagulable State	2.94 (1.69-5.13)	<.001	1.09 (0.58-2.05)	0.78
Rivaroxaban vs Aspirin	1.83 (1.41-2.36)	<.001	1.38 (1.20-1.60)	<.001
Apixaban vs Aspirin	1.30 (1.00-1.68)	0.05	1.20 (1.04-1.39)	0.01
Enoxaparin vs Aspirin	2.02 (1.51-2.71)	<.001	1.22 (1.01-1.46)	0.03
Warfarin vs Aspirin	1.39 (1.02-1.90)	0.04	1.42 (1.19-1.68)	<.001

Logistic regression adjusted for age, sex, length of stay, inpatient vs. outpatient, knee vs. hip arthroplasty, obesity, cancer, chronic kidney disease, history of venous thromboembolism, antiphospholipid antibody syndrome, hereditary hypercoagulable state (factor V Leiden, protein C or S deficiency, prothrombin gene mutation, and antithrombin gene mutation). ALPA: Antiphospholipid Antibody.

© 2023 Simon SJ et al. *JAMA Network Open.*

eFigure 1. Box plot of propensity scores for receiving a direct oral anticoagulant (DOAC) by DOAC and aspirin prior to matching.

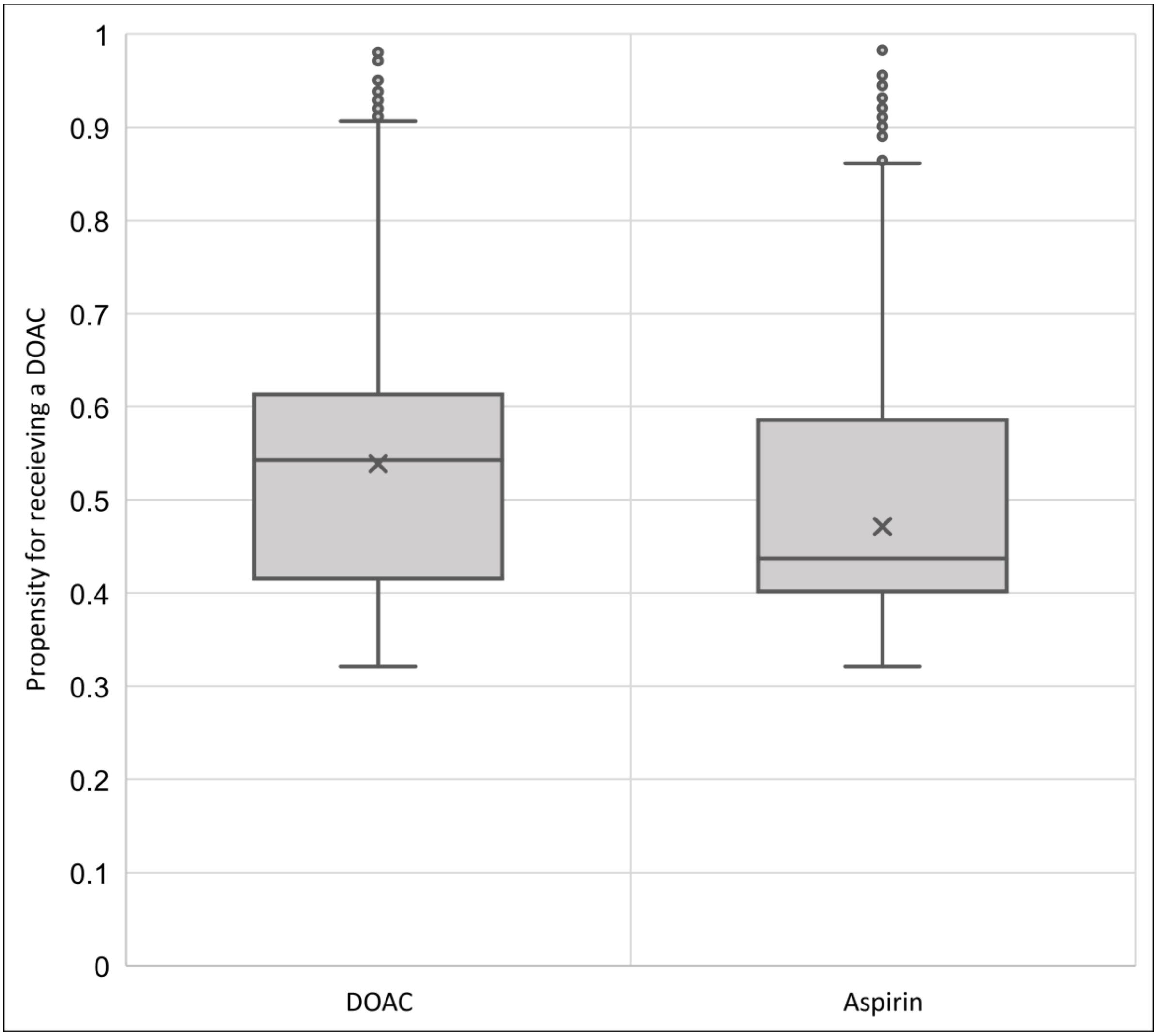

© 2023 Simon SJ et al. *JAMA Network Open.*

eTable 7. Overview of patient and surgical variables of the propensity matched cohort. DOAC: direct oral anticoagulant.

Variables	Total<br>No. (%)	Aspirin<br>No. (%)	DOAC<br>No. (%)
Total	13250 (84.5)	7844 (50.0)	7844 (50.0)
Age: < 65	2438 (15.5)	6625 (84.5)	6625 (84.5)
Age: ≥ 65	6692 (42.7)	1219 (15.5)	1219 (15.5)
Male	8996 (57.3)	3346 (42.7)	3346 (42.7)
Female	118 (0.8)	4498 (57.3)	4498 (57.3)
History of Venous Thromboembolism	118 (0.8)	59 (0.8)	59 (0.8)
Obese	610 (3.9)	59 (0.8)	59 (0.8)
Cancer	470 (3.0)	305 (3.9)	305 (3.9)
Chronic Kidney Disease	8 (0.1)	235 (3.0)	235 (3.0)
Antithrombin or Prothrombin Gene Mutation	9 (0.1)	6 (0.1)	2 (0.03)
Factor V Leiden	14 (0.1)	3 (0.04)	6 (0.1)
Protein C or S Deficiency	76 (0.5)	8 (0.1)	6 (0.1)
Antiphospholipid Antibody or Lupus Anticoagulant	5622 (35.8)	38 (0.5)	38 (0.5)
Hip Arthroplasty	10066 (64.2)	2811 (35.8)	2811 (35.8)
Knee Arthroplasty	778 (5.0)	5033 (64.2)	5033 (64.2)
Outpatient	14854 (94.7)	385 (4.9)	393 (5.0)
Inpatient	5494 (35.0)	7428 (94.7)	7426 (94.7)
Ambulatory Surgical Center	6312 (40.2)	31 (0.4)	25 (0.3)
Surgery Year: 2017	3882 (24.8)	2414 (30.8)	3080 (39.3)
Surgery Year: 2018	2723 (17.4)	3109 (39.6)	3203 (40.8)
Surgery Year: 2019	5293 (33.7)	2321 (29.6)	1561 (19.9)
Length of Day: 0 Days	7311 (46.6)	1332 (17.0)	1391 (17.7)
Length of Stay: 1-2 Days	361 (2.3)	2676 (34.1)	2617 (33.4)
Length of Stay: 3-4 Days	13250 (84.5)	3708 (47.3)	3603 (45.9)
Length of Stay: ≥ 5 Days	2438 (15.5)	128 (1.6)	233 (3.0)

© 2023 Simon SJ et al. *JAMA Network Open.*

eFigure 2. Absolute standardized mean differences between direct oral anticoagulant and aspirin patients. Orange dots represent the whole cohort and blue dots represent the propensity matched cohort.

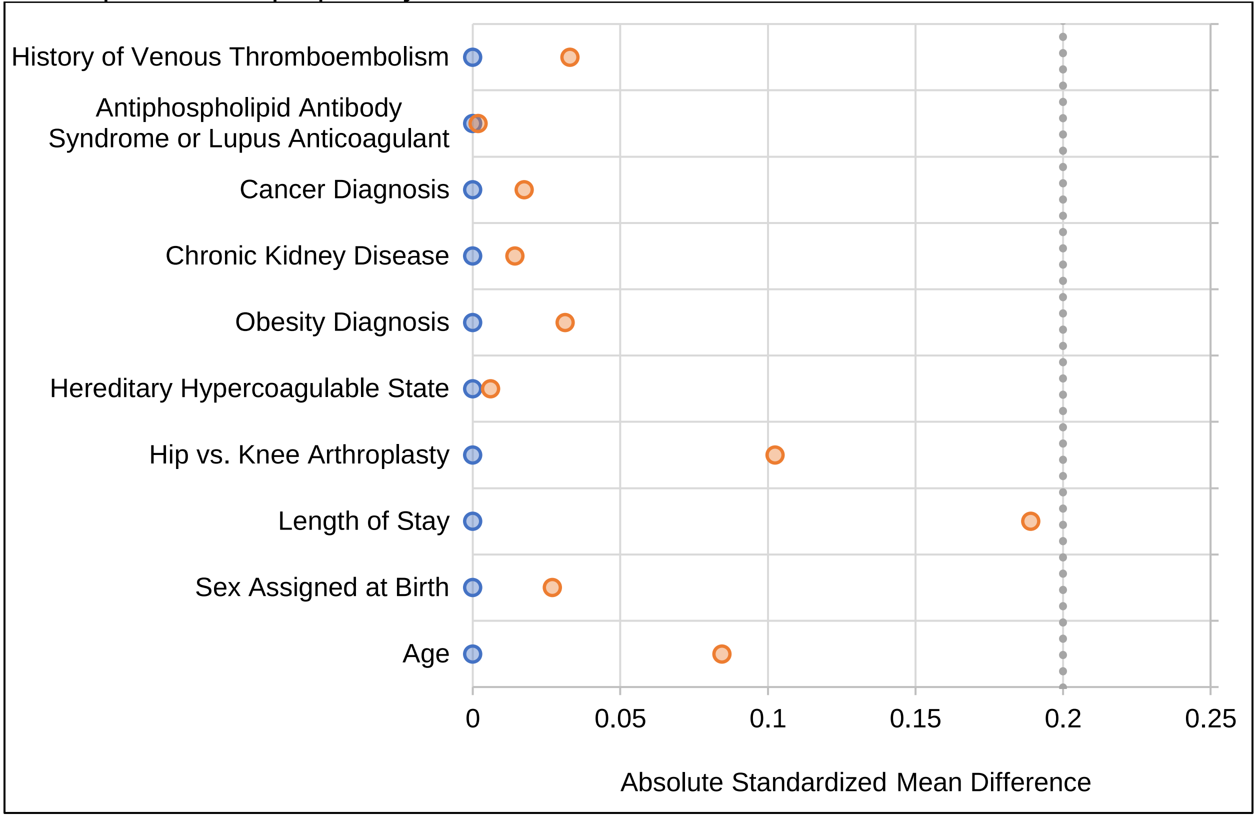

© 2023 Simon SJ et al. *JAMA Network Open.*
