# Supplementary material for: Venous Thromboembolism in Total Hip and Total Knee Arthroplasty
Source: JAMA Netw Open. 2023 Dec 1;6(12):e2345883. doi: 10.1001/jamanetworkopen.2023.45883 (PMC10692868; doi:10.1001/jamanetworkopen.2023.45883)
Supplement: Supplement 2. — Data Sharing Statement [file jamanetwopen-e2345883-s002.pdf]

Data Sharing Statement

Simon. Venous Thromboembolism in Total Hip and Total Knee Arthroplasty. *JAMA Netw Open*. Published December 01, 2023. doi:10.1001/jamanetworkopen.2023.45883

Data
Data available: No

Additional Information
Explanation for why data not available: Data may be obtained from a third party and are not publicly available. The IBM MarketScan Commercial Claims data that support the findings of this study are available from IBM MarketScan Research Databases, but restrictions apply to the availability of these data, which were used under license for the current study, and so are not publicly available.
